# Supplementary material for: All-ferroelectric implementation of reservoir computing
Source: Nat Commun. 2023 Jun 16;14:3585. doi: 10.1038/s41467-023-39371-y (PMC10275999; doi:10.1038/s41467-023-39371-y)
Supplement: Supplementary file 1 — Supplementary Information [file 41467_2023_39371_MOESM1_ESM.pdf]

Supplementary Information

All-ferroelectric implementation of reservoir computing

Zhiwei Chen[1], Wenjie Li[1], Zhen Fan[1]*, Shuai Dong[1], Yihong Chen[1], Minghui Qin[1], Min Zeng[1], Xubing Lu[1], Guofu Zhou[2], Xingsen Gao[1] & Jun-Ming Liu[1,3]

[1] *Institute for Advanced Materials and Guangdong Provincial Key Laboratory of Optical Information Materials and Technology, South China Academy of Advanced Optoelectronics, South China Normal University, Guangzhou 510006, China*

[2] *National Center for International Research on Green Optoelectronics, South China Normal University, Guangzhou 510006, China*

[3] *Laboratory of Solid State Microstructures and Innovation Center of Advanced Microstructures, Nanjing University, Nanjing 210093, China*

*Email: fanzhen@m.scnu.edu.cn

This Supplementary Information file includes:

Supplementary Figure S1-S33

Supplementary Table S1-S2

Supplementary Note 1-4

Supplementary References

Table S1. Comparison of the key features of the ferroelectric-based RC systems reported in recent works and ours.

Reservoir system	Reservoir			Readout network	Circuit-level demonstration?	Power consumption (for device in reservoir only)	Accuracy (MNIST)
	Devices	Polarization dynamics clearly revealed?	Richness of polarization dynamics				
Ref. [1]	$Hf_{0.5}Zr_{0.5}O_2$-based FTJ	No	Medium	RRAM	Yes	~70 μW	92.3%
Ref. [2]	$Hf_{0.5}Zr_{0.5}O_2$-based FeFET	No	Low	Simulation	No	~900 μW	–
Ref. [3]	$Hf_{0.5}Zr_{0.5}O_2$-based FeFET (volatile)	No	Medium	$Hf_{0.5}Zr_{0.5}O_2$-based FeFET (volatile)	No	~3000 μW	95.1%
Ref. [4,5]	α-In_2Se_3-based FeFET	No	Medium	Simulation	No	~60 μW	86.1%
Ours	$BiFeO_3$-based FD (volatile)	Yes	High	$BiFeO_3$-based FD (nonvolatile)	Yes	~11.8 μW	89.5%

Based on Table S1, two facts regarding the existing ferroelectric-based RC systems can be extracted:

(i) The ferroelectric-based RC systems in most of the recent works used ferroelectric tunnel junctions (FTJs) and ferroelectric field-effect transistors (FeFETs) to implement only the reservoirs, while the readout networks were either simulated or implemented with a mature RRAM chip. Only the work in Ref. [3] attempted to use a $Hf_{0.5}Zr_{0.5}O_2$ (HZO)-based FeFET to implement the readout network, but this FeFET, identical that used for the reservoir, was indeed volatile and failed to meet the functional requirements of the readout network.

(ii) The ferroelectric-based RC systems in most of the recent works were demonstrated at the device level. Only the work in Ref. [1] reported a circuit-level demonstration, but the readout network was implemented with RRAM rather than ferroelectric devices.

From the above two facts, it is noted that so far there has been no demonstration of an all-ferroelectric RC system (particularly at the circuit level). This is disappointing because the all-ferroelectric RC system promises higher performance and robustness than the existing RRAM-based RC systems (see reasons in the Introduction section of the main text). In this work, we develop an all-ferroelectric RC system, where the reservoir and readout network are implemented with the volatile and nonvolatile FDs, respectively, and demonstrate its temporal signal processing capability at the circuit level (albeit with simple tasks). Therefore, our work indeed realizes an all-ferroelectric RC system for the first time.

Table S1 also reveals several advantages of our all-ferroelectric RC system, as summarized below:

(a) The use of FDs as building blocks

As shown in Table S1, only our work uses FDs as the building blocks of an RC system, while previous works used FTJs and FeFETs. As explained in detail in the Introduction section of the main text, FTJ and FeFET possess inherently large depolarization fields (E_{dp}s), making them voluntary to exhibit volatile characteristics while difficult to be engineered into nonvolatile memristors to implement the readout network. By contrast, FD is inherently subjected to a much smaller E_{dp}, and hence it can readily function as a nonvolatile memristor. In addition, by judiciously introducing an E_{imp} without changing

the device structure, the FD is engineered to be volatile. The volatile and nonvolatile FDs are further used to implement the reservoir and readout network, respectively, eventually forming a well-functioning RC system.

Using FDs as the building blocks is therefore the key to the successful implementation of the all-ferroelectric RC system, which would provide great inspiration for researchers working on ferroelectric-based neuromorphic computing. Additionally, it is noteworthy that the derivation of the volatile and nonvolatile FDs from the same device structure has not been realized in other types of ferroelectric memristors.

(b) Clearly revealed polarization dynamics and high richness of polarization dynamics

Table S1 highlights that the polarization dynamics of our volatile FD are clearly revealed. However, the polarization dynamics of previously used FTJs and FeFETs (for reservoirs) remain largely unclear, because it is difficult or even impractical to directly measure polarization in these devices owing to either large leakage current or poor charge screening. By contrast, FD allows the direct polarization measurement because of the suppressed leakage current (arising from the relatively thick ferroelectric film and the reverse-biased Schottky barrier) and the good charge screening provided by the metal electrodes [6]. Polarization dynamics of our volatile FD have thus been systematically studied [Figures 2(b) and 3(b) in the main text and Figures S4, S5(a)-(c), S9, and S14(a)-(b)]. In particular, the E_{imp}-induced polarization back-switching is clearly revealed in our volatile FD [Figure 3(b) in the main text and Figure S9], which could greatly enrich the polarization dynamics.

Table S1 also presents that the richness of polarization dynamics of our volatile FD is in principle the highest, as explained as follows. For the $Hf_{0.5}Zr_{0.5}O_2$ (HZO)-based FeFET [2], it exhibits only the nonlinear, history-dependent polarization switching behavior, which is common for all the ferroelectric devices. It therefore has the lowest richness of polarization dynamics. For other FTJs [1] and FeFETs [3-5], although the E_{dp}-induced polarization decay was claimed to exist in these devices, it was not unambiguously revealed because of the difficulty in directly measuring the polarization, as mentioned earlier. Even if the E_{dp} existed, it was only able to reduce the polarization toward zero, while unable to reverse the direction of polarization. For our volatile FD, the presence of E_{imp} and the E_{imp}-induced polarization back-switching are clearly revealed. Moreover, E_{imp} could even reverse the direction of polarization [Figure S9(c)]. Therefore, the richness of polarization dynamics of our volatile FD is in principle higher than those of previous devices with E_{dp}.

(c) Low power consumption

Table S1 compares the power consumptions of different ferroelectric memristors used for reservoirs. Our volatile FD exhibits the lowest power consumption of ~11.8 μW, well due to the relatively low operation voltage and suppressed leakage current, as mentioned earlier. Such power consumption is even at least 3 times lower than those of the state-of-the-art filamentary memristors for reservoirs [7-9]. Also note that the power consumption of our nonvolatile FD for readout network is even lower, reaching ~140 nW. The details about the power consumption estimation can be found in Supplementary Note 4.

(d) High prediction performance

In terms of the prediction performance, although the counterparts in Ref. [1] and [3] achieve higher accuracies than our all-ferroelectric RC system in the MNIST handwritten digit recognition task (see Table S1), they use either multilayer readout networks [1] or more complex pre-processing [3], making the direct comparison between these accuracies unfair. In fact, the 89.5% accuracy achieved by our all-ferroelectric RC system is 6.5% higher than that achieved by a pioneering diffusive memristor-based RC system [10], whose image pre-processing approach, reservoir architecture, and readout network size are similar to ours.

In addition, our all-ferroelectric RC system achieves an ultralow NRMSE value of 0.017 in the Hénon map time-series prediction (see Figure 6 in the main text), which also underscores its high prediction performance.

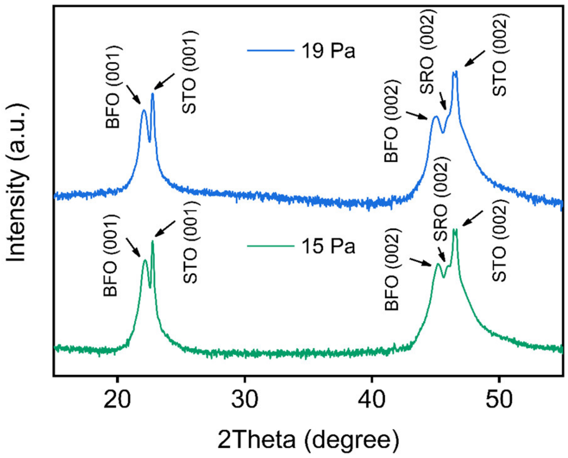

Figure S1. XRD θ-2θ scans of the BFO films grown on the SRO-buffered (001)-oriented STO substrates under oxygen pressures of 15 Pa and 19 Pa.

Only (00*l*) diffraction peaks from BFO, SRO, and STO are observed in both of the two BFO films, evidencing their phase purity. In addition, according to the positions of the BFO (00*l*) peaks, one can conclude that both BFO films exhibit a rhombohedral-like phase [11].

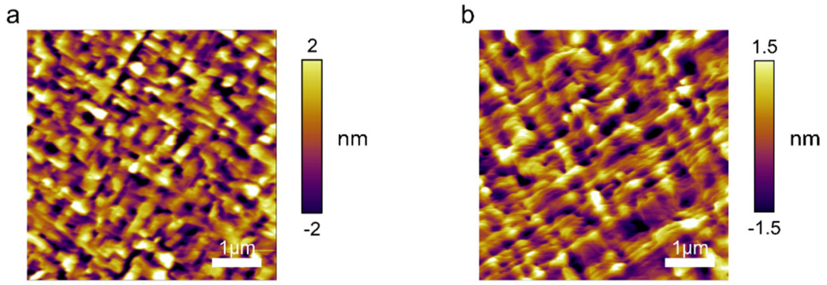

Figure S2. AFM topography images of the BFO films grown under oxygen pressures of (a) 15 Pa and (b) 19 Pa.

Both of the BFO films exhibit a step-bunching morphology, which is a typical feature of relatively thick epitaxial BFO films grown on SRO-buffered (001)-oriented STO substrates with no miscut [12]. In addition, the surfaces of both films are rather flat with root-mean-square roughness values smaller than ~1.2 nm.

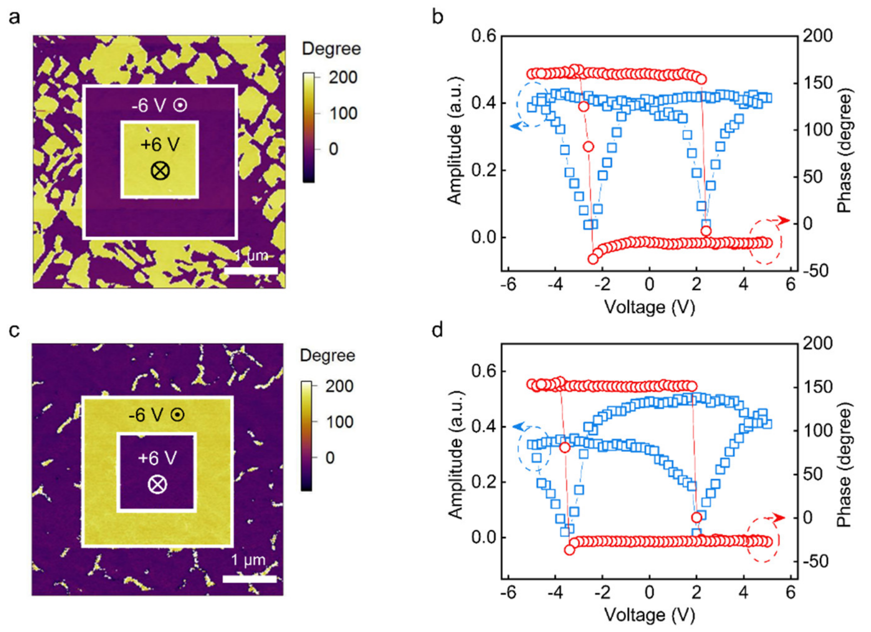

Figure S3. PFM (a,c) phase images after the box-in-box writing (outer: −6 V; inner: 6 V) and (b,d) amplitude and phase hysteresis loops for (a,b) 15 Pa and (c,d) 19 Pa BFO films.

Figures S3(a) and (c) show the PFM phase images after the box-in-box writing on the 15 Pa and 19 Pa BFO films, respectively. It is observed that the domains in both films can be reversibly switched, verifying their ferroelectricity. However, the domain configurations in the as-grown regions of the two films are different. In the 19 Pa film, most of the as-grown regions are occupied by downward domains, while there are only a few tiny and irregularly-shaped upward domains. The dominance of downward domains may suggest that a downward E_{imp} exists in the 19 Pa film. By contrast, in the 15 Pa film, the upward domains in the as-grown regions become much bigger, and their total area is comparable to that of the downward domains. This suggests that the E_{imp} becomes negligible in the 15 Pa film.

Figures S3(b) and (d) present the PFM amplitude and phase hysteresis loops of the 15 Pa and 19 Pa BFO films, respectively. Both films exhibit butterfly-like amplitude loops and square phase loops with 180° switching. However, while the amplitude and phase loops of the 15 Pa film are rather symmetric with respect to 0 V, those of the 19 Pa film exhibit apparent negative voltage offsets. This suggests again that a downward E_{imp} exists in the 19 Pa film while it is absent in the 15 Pa film.

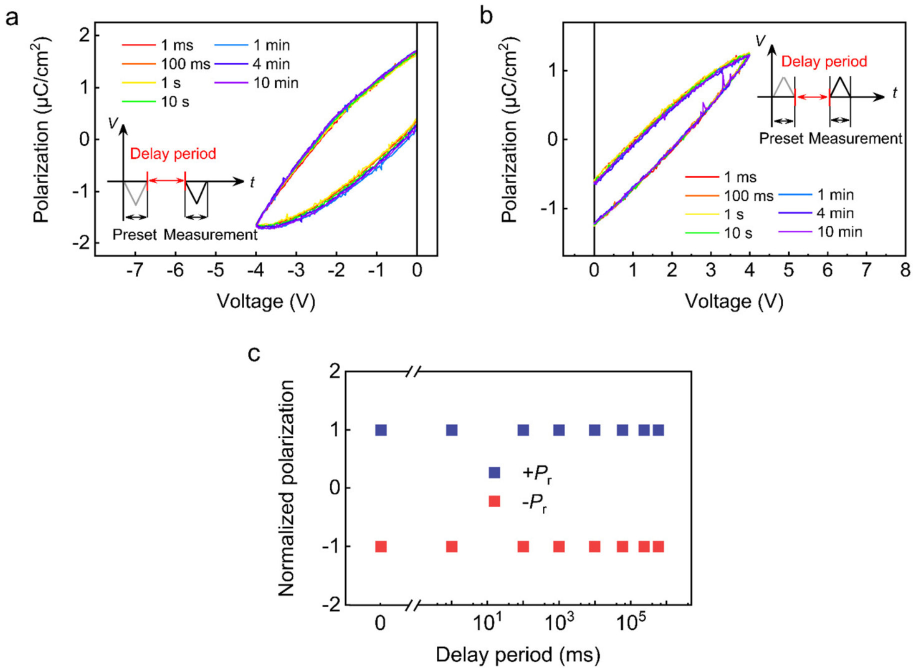

Figure S4. Polarization retention behavior of the Pt/BFO (15 Pa)/SRO nonvolatile FD. (a) Negative and (b) positive monopolar *P*-*V* loops measured with different delay periods. Insets in a and b show the schematics of the pulses applied in the monopolar loop measurements. (c) Normalized $\pm P_r$ as a function of the delay period.

As shown in the inset of Figure S4(a) [Figure S4(b)], the device is first set in the P_{up} (P_{down}) state by applying a negative (positive) preset pulse, and after a delay period the polarization state is probed by applying a negative (positive) measurement pulse. If certain polarization switches back to the P_{down} (P_{up}) direction during the delay period, it could be switched again to the P_{up} (P_{down}) direction by the negative (positive) measurement pulse. Therefore, the back-switched polarization can be reflected by the switched polarization measured by the measurement pulse.

Figures S4(a) and (b) show that both the negative and positive monopolar *P*-*V* loops exhibit very small hysteresis windows and they almost do not change with increasing delay period, suggesting that almost no polarization back-switching occurs during the delay period for both P_{up} and P_{down} states.

Furthermore, the results in Figures S4(a) and (b) can be used to derive the temporal evolutions of remnant polarizations, i.e., $-P_r$ and $+P_r$, as shown in Figure S4(c). One can see that both $-P_r$ and $+P_r$ remain almost unchanged with increasing delay period, confirming the good stability of both P_{up} and P_{down} states.

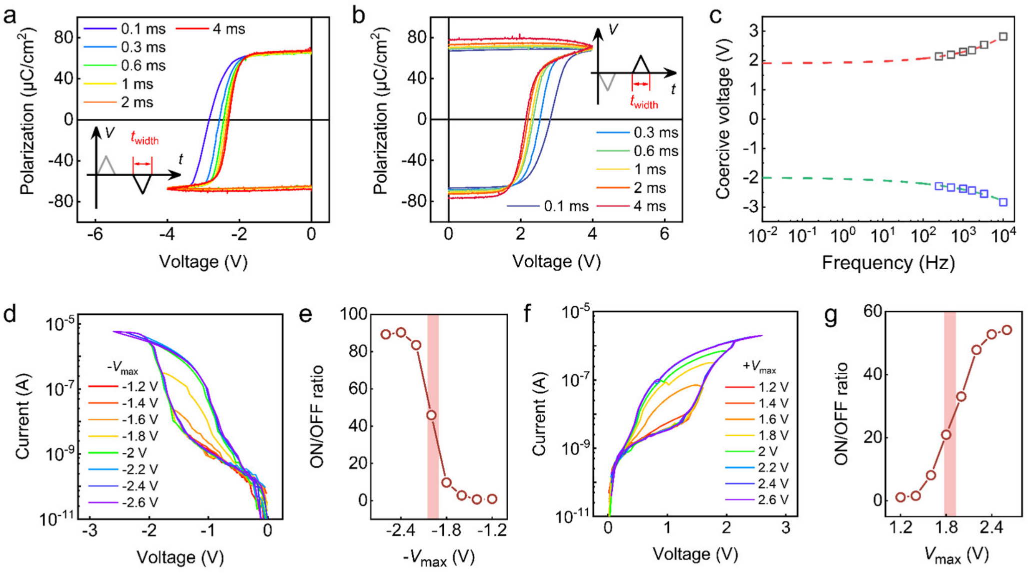

Figure S5. Correlation between polarization switching and resistive switching in the nonvolatile FD. (a) Negative and (b) positive monopolar *P-V* loops at different pulse widths. (c) Positive and negative coercive voltages as a function of frequency (i.e., the reciprocal of pulse width). Hysteretic *I-V* curves measured by applying the voltage sweeps of (d) $0 \rightarrow -V_{max} \rightarrow 0$ ($-V_{max}$ varies from −1.2 V to −2.6 V) and (f) $0 \rightarrow +V_{max} \rightarrow 0$ ($+V_{max}$ varies from 1.2 V to 2.6 V). ON/OFF ratios, read at −1 V and 1 V, respectively, as a function of (e) $-V_{max}$ and (g) $+V_{max}$. Data in e and g are extracted from d and f, respectively. In a and d (b and f), the device is set in the initial P_{down} (P_{up}) state before each measurement.

Figures S5(a-c) show that both positive and negative coercive voltages decrease in magnitude as the frequency decreases. By extrapolating the coercive voltage-frequency curves, the positive and negative coercive voltages in the low-frequency region (e.g., on the order of 10^{-2} Hz) are roughly estimated as ~1.9 and ~−2 V, respectively.

Note that the *I*-*V* curves [Figures 2(c) and 3(c) in the main text and Figures S5(d) and (f)] were measured in the DC voltage sweep mode with a very low frequency (on the order of 10^{-2} Hz). It is thus assumed that in the DC voltage sweep mode the up-to-down and down-to-up polarization switching events occur at ~1.9 and ~−2 V, respectively.

On the other hand, the critical voltage where HRS switches to LRS may be defined as the $-V_{max}$ (or $+V_{max}$) which leads to the abrupt increase in ON/OFF ratio. For the HRS → LRS switching in the negative (positive) voltage region, as shown in Figure S5(e) [Figure S5(g)], the most abrupt increase in ON/OFF ratio is observed at ~−2 V (~1.9 V). These negative and positive critical voltages for resistive switching well correspond to the negative and positive low-frequency coercive voltages for polarization switching, respectively, suggesting that the resistive switching is induced by the polarization switching.

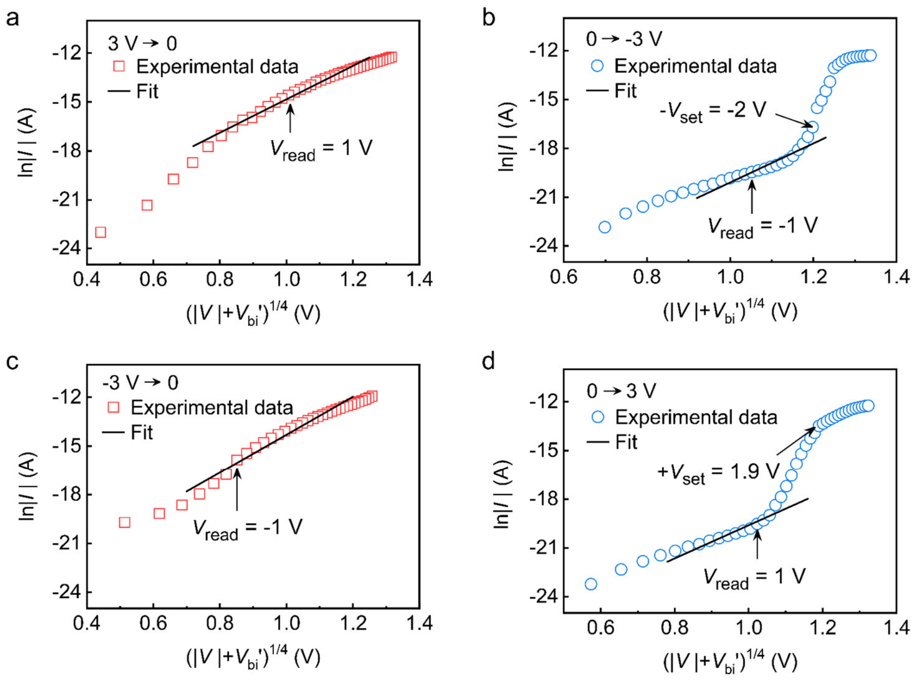

Figure S6. Conduction mechanism analysis for the nonvolatile FD. ln|I| versus $(|V|+V_{bi}')^{1/4}$ curves, as replotted from the *I-V* curves in Figure 2(c) in the main text, along the voltage sweeps of (a) +3 V → 0, (b) 0 → −3 V, (c) −3 V → 0, and (d) 0 → +3V. The read voltages and the critical voltages for resistive switching are denoted as V_{read} and $\pm V_{set}$, respectively, in each panel.

As seen from Figure 2(c) in the main text, the device in either P_{up} or P_{down} state exhibits asymmetric *I-V* curves. For example, as the voltage sweeps from +3 V to 0 and then to a negative voltage below the critical voltage $-V_{set}$, the device is in the P_{down} state and the *I-V* curves in the positive and negative voltage regions are asymmetric. This suggests that the dominant conduction mechanism in the device is interface-limited rather than bulk-limited. Two typical interface-limited conduction models, namely, Schottky emission and Fowler–Nordheim (FN) tunneling, were mainly considered in this work.

The FN tunneling was first attempted for fitting (results not shown). However, the fitted values of barrier heights are unreasonably small (on the order of 0.001 eV), thus excluding the FN tunneling as the dominant conduction mechanism in the Pt/BFO/SRO device.

Then, the *I-V* curves were fitted by using the Schottky emission model given below [13]:

$$\ln(I)=\left[\ln(AA^{*}T^{2})-\frac{q}{kT}\Phi_{\mathrm{B}}^{0}\right]+\frac{q}{kT}\left(\frac{q^{3}N_{\mathrm{eff}}}{8\pi^{2}\varepsilon_{0}^{3}\varepsilon_{\mathrm{op}}^{2}\varepsilon_{\mathrm{st}}}\right)^{\frac{1}{4}}\left(V+V_{\mathrm{bi}}'\right)^{\frac{1}{4}}, \tag{S1}$$

where A is the electrode area, A^{*} is the Richardson constant, T is the absolute temperature, q is the electron charge, k is the Boltzmann constant, N_{eff} is the effective charge density, ε_0 is the vacuum permittivity, $\varepsilon_{\mathrm{op}}$ and $\varepsilon_{\mathrm{st}}$ are the optical and static dielectric constants of the ferroelectric layer, respectively, and V_{bi}' is the apparent built-in potential. Here, the $\varepsilon_{\mathrm{op}}$ and $\varepsilon_{\mathrm{st}}$ values of BFO used for fitting are 6.25 [14] and 60 [15], respectively.

The fitting results are shown as the black lines in Figure S6. It is seen that all the $\ln|I|$-$(|V|+V_{\mathrm{bi}}')^{1/4}$ curves agree well with the fitting lines in certain voltage ranges of interest. Here, the voltage range of interest covers the voltages that are around the read voltage (e.g., ±1 V) and below the critical voltage for resistive switching. The good fits suggest that for the device in both P_{up} and P_{down} states the Schottky emission may be the dominant conduction mechanism in these voltage ranges.

The BFO film is typically a *p*-type semiconductor due to the Bi loss [16], and the *p*-type character of our BFO film has been demonstrated recently [6]. Note that the BFO film can still exhibit *p*-type character despite the existence of oxygen vacancies probably

because of the dominance of Bi loss. In addition, BFO typically has an electron affinity of ~3.3 eV and a bandgap of ~2.7 eV [17,18], while Pt and SRO have work functions of ~5.3 eV and ~5.2 eV, respectively. Based on these parameters, *p*-type Schottky barriers may be formed at both the Pt/BFO and BFO/SRO interfaces. The BFO/SRO and Pt/BFO barriers are the current-limiting barriers under negative and positive voltages, respectively.

Based on the intercepts of the fitting lines on the $\ln|I|$ axis, the heights of the BFO/SRO and Pt/BFO barriers can be extracted. Specifically, using the intercepts in Figure S6(b) and (c), the heights of the BFO/SRO barrier are extracted to be ~1.00 eV and ~0.84 eV in the P_{down} and P_{up} states, respectively (note that the device is in the P_{down} and P_{up} states during the voltage sweeps of $0 \rightarrow -V_{\mathrm{set}}$ and -3 V $\rightarrow 0$, respectively). Similarly, based on Figures S6(a) and (d), the heights of the Pt/BFO barrier are extracted to be ~0.82 eV and ~0.94 eV in the P_{down} and P_{up} states, respectively. These fitting results evidence that the polarization can modulate the Schottky barrier height, in turn modifying the conductance. The resistive switching mechanism will be described in more detail with Figure S7.

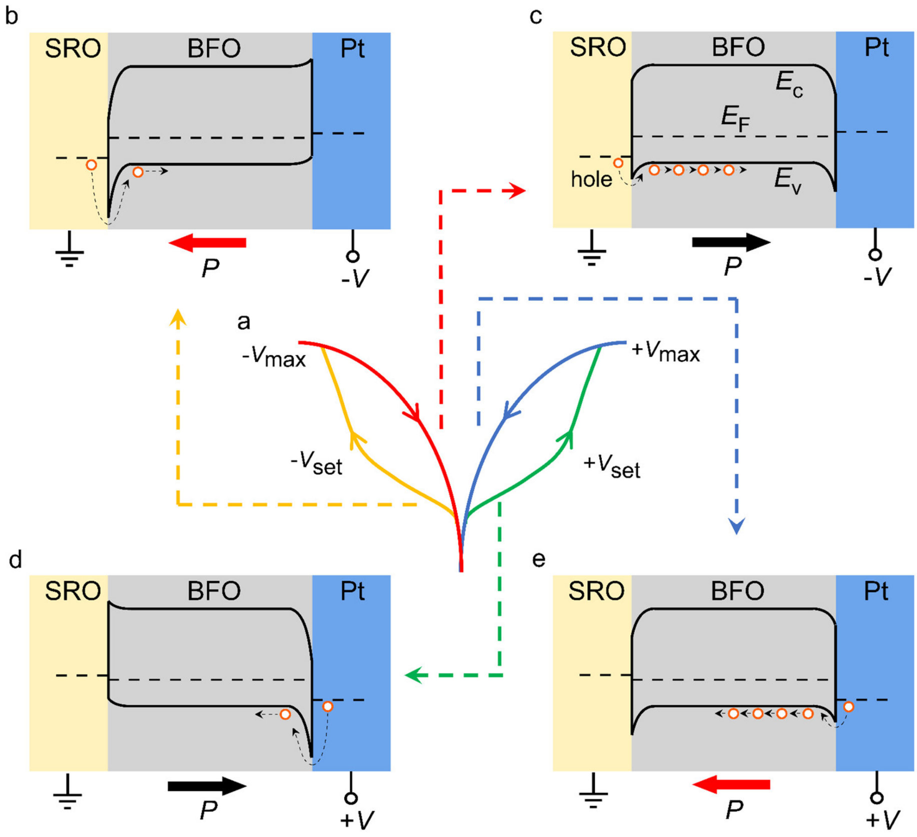

Figure S7. Resistive switching mechanism in the nonvolatile FD. (a) Schematic hysteretic *I-V* curves representing the switchable diode-type resistive switching behavior. The negative and positive critical voltages where HRS switches to LRS are indicated by $-V_{set}$ and $+V_{set}$, respectively, while the maximum negative and positive applied voltages are indicated by $-V_{max}$ and $+V_{max}$, respectively. Schematics showing the energy band diagrams and conduction processes (b) under a small negative voltage in the P_{down} state, (c) under a small negative voltage in the P_{up} state, (d) under a small positive voltage in the P_{up} state, and (e) under a small positive voltage in the P_{down} state.

The fitting results shown in Figure S6 have demonstrated that the conduction in the Pt/BFO/SRO nonvolatile FD is dominated by the Schottky emission and the polarization

switching-induced barrier height change causes the resistive switching. We can now illustrate the resistive switching mechanism in more detail by using the energy band diagrams.

As mentioned earlier, our BFO film is a *p*-type semiconductor and two *p*-type Schottky barriers may be formed at both the Pt/BFO and BFO/SRO interfaces. Given that the device is subjected to an initial voltage sweep of $+V_{max} \rightarrow 0$, it is thus set in the initial P_{down} state. Then, as the voltage sweeps from 0 to $-V_{set}$, the BFO/SRO barrier is reverse-biased and thus limits the hole injection. Because the positive polarization charge at the BFO/SRO barrier enhances the barrier height, the HRS is obtained [see Figure S7(b)]. As the negative voltage exceeds $-V_{set}$, the polarization switches to the upward direction. This P_{up} state can be maintained as the voltage sweeps from $-V_{max}$ back to 0. The negative polarization charge at the BFO/SRO barrier lowers the barrier height, thus leading to the HRS → LRS switching [see Figure S7(c)].

As the applied voltage changes to positive (below $+V_{set}$), the device is still in the P_{up} state. Meanwhile, the Pt/BFO barrier is reverse-biased and thus limits the hole injection. The Pt/BFO barrier is now relatively high due to the positive polarization charge located there, resulting in the HRS [Figure S7(d)]. As the applied voltage exceeds $+V_{set}$, the polarization switching occurs. The negative polarization charge at the Pt/BFO barrier lowers the barrier height, causing the HRS → LRS switching [see Figure S7(e)].

Therefore, the mechanism for the switchable diode-type resistive switching has been well explained, and it agrees with those reported previously [16,19]. Also note that the polarization switching-induced barrier height modulation, i.e., the key to the proposed resistive switching mechanism, has been proved by the fitting results in Figure S6.

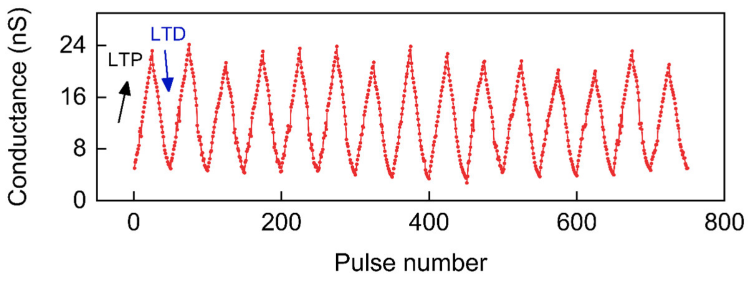

Figure S8. Multi-cycle LTP/LTD characteristics.

The multi-cycle LTP/LTD characteristics were measured with a device which was different from that used in Figure 2(d) in the main text. Due to the D2D variation, the conductance values shown in Figure S8 are different from those shown in Figure 2(d) in the main text. Nevertheless, Figure S8 demonstrates that the LTP and LTD processes can be repeated for multiple cycles.

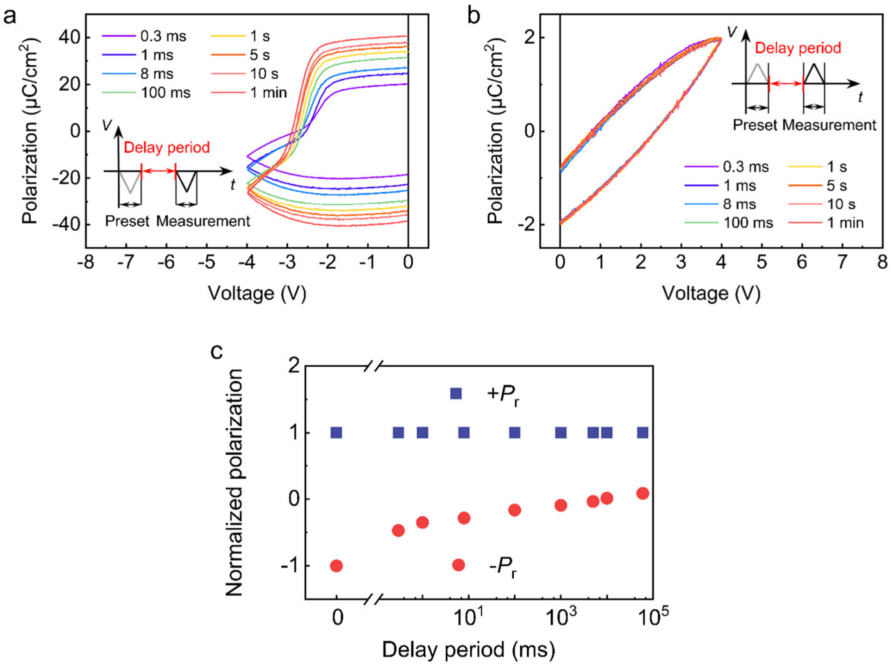

Figure S9. Polarization retention behavior of the Pt/BFO (19 Pa)/SRO volatile FD. (a) Negative and (b) positive monopolar *P*-*V* loops measured with different delay periods. Insets in a and b show the schematics of the pulses applied in the monopolar loop measurements. (c) Normalized $\pm P_r$ as a function of the delay period.

The method of the polarization retention measurement has been described in Figure S4. Figure S9(a) shows that all the negative monopolar *P*-*V* loops exhibit an "S" shape, which is a fingerprint of polarization switching. Moreover, the switched polarization becomes larger with increasing delay period. It is therefore deduced that the polarization back-switching occurs in the P_{up} state, and the back-switched polarization increases with the delay period. By contrast, Figure S9(b) shows that the positive monopolar *P*-*V* loops at different delay periods all exhibit very small hysteresis windows and they almost overlap, suggesting that almost no polarization back-switching occurs in the P_{down} state.

We further plot the $\pm P_r$ as a function of delay period in Figure S9(c). It is seen that $+P_r$ almost does not change with increasing delay period, while $-P_r$ gradually decreases and eventually changes its sign as the delay period increases. This unambiguously confirms the existence of the downward E_{imp}, which causes the polarization back-switching only in the P_{up} state. This also excludes the depolarization field (E_{dp}) as the major driving force for the polarization back-switching, because E_{dp} would induce the polarization back-switching in both P_{up} and P_{down} states.

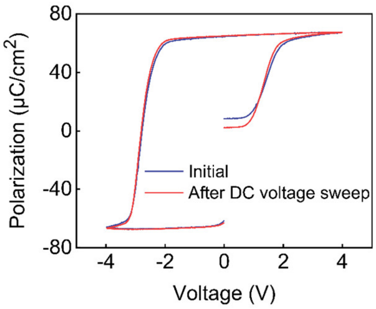

Figure S10. *P*-*V* loops of the Pt/BFO (19 Pa)/SRO device before and after applying a DC voltage sweep of 3 V → −3 V → 3 V to it.

First, the presence of negative voltage offset and gap in the *P*-*V* loop already demonstrates that there is a downward E_{imp} and this E_{imp} is persistent against the voltage pulse; otherwise, the *P*-*V* loop would not show such voltage offset and gap. Second, as shown in Figure S10, the *P*-*V* loop almost does not change after applying a DC voltage sweep to the device, suggesting that the E_{imp} remains almost unchanged after applying the DC voltage sweep.

It is therefore concluded that the E_{imp} is stable against electric field (including both pulsed and DC electric fields) at room temperature. Note that this conclusion is valid throughout this study because the pulsed and DC voltages applied in this study are no larger than ±4 V and ±3 V, respectively. Further enhancing the electric field may invalidate this conclusion, which is out of the scope of this study.

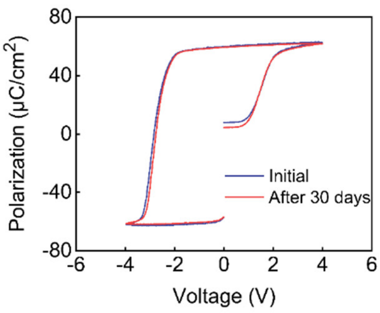

Figure S11. *P-V* loops of the Pt/BFO (19 Pa)/SRO device before and after leaving it in the ambient air at room temperature for 30 days.

The *P-V* loops of the device before and after a 30-day exposure to the ambient air almost overlap, suggesting that the E_{imp} almost does not change after a long duration. It is therefore concluded that the E_{imp} is stable over time at room temperature.

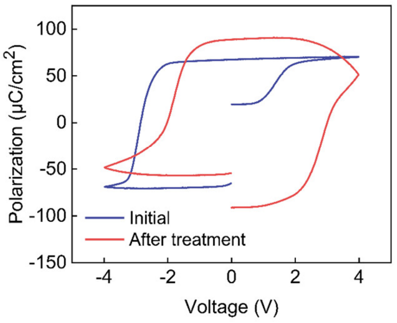

Figure S12. *P-V* loops of the Pt/BFO (19 Pa)/SRO device before and after the thermal treatment, where the device was set to the P_{up} state and then annealed at 250 °C for 60 min in air.

To evaluate the temperature stability of E_{imp}, the initial *P-V* loop of the Pt/BFO (19 Pa)/SRO device was first measured at room temperature. Then, the device was set to the P_{up} state and then annealed at 250 °C for 60 min in air. After this treatment, the *P-V* loop exhibits a positive voltage offset (see Figure S12), suggesting that the direction of E_{imp} is now pointing upward, opposite to that of the initial E_{imp}.

Therefore, E_{imp} can be changed at high temperature. Nevertheless, because all the electrical measurements in this study were performed at room temperature, the downward E_{imp} in the 19 Pa film can be considered as relatively stable.

Note that the origin for E_{imp} and why E_{imp} changes at high temperature are explained in the discussion on Figure S13.

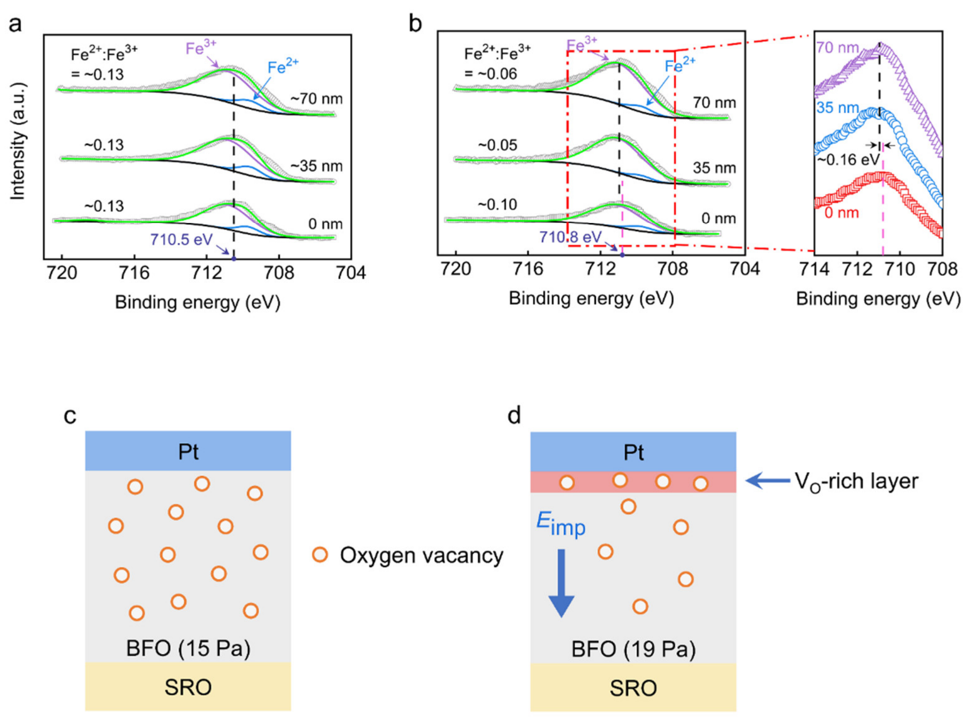

Figure S13. Depth-dependent XPS study. XPS spectra of Fe $2p_{3/2}$ measured at different depths (0, ~35, and ~70 nm) of the (a) 15 Pa and (b) 19 Pa BFO films. Inset in b shows an enlarged view of the peaks. Schematics showing the distributions of oxygen vacancies in the (c) 15 Pa and (d) 19 Pa BFO films.

The XPS results can be interpreted as follows.

(a) Different amounts and distributions of oxygen vacancies in the 15 Pa and 19 Pa films

Figure S13(a) shows that the Fe $2p_{3/2}$ peaks measured at different depths of the 15 Pa film have almost the same binding energy, suggesting that the Fe valence does not change much as the depth varies. However, as shown in Figure S13(b), the Fe $2p_{3/2}$ peak measured at the surface (i.e., at the 0 nm depth) of the 19 Pa film is shifted by ~0.16 eV toward lower

binding energy relative to those measured in the bulk regions (i.e., at the ~35 and ~70 nm depths). This peak shift is indeed not very small considering that the upper limit of the peak shift (i.e., the binding energy difference between the Fe^{2+} and Fe^{3+} $2p_{3/2}$ peaks) is only ~1.3 eV [20]. In addition, this peak shift is still greater than the energy resolution of our XPS system, i.e., 0.05 eV [21,22]. It may thus be safe to deduce that the Fe valence is lower at the surface than in the bulk regions.

By further comparing Figures S13(a) and (b), it is revealed that the average peak position of the 15 Pa film is at a lower binding energy compared with that of the 19 Pa film, implying that the average Fe valance of the former is lower than that of the latter.

The above results regarding the Fe valance can be used to extract the information of oxygen vacancies. To do this, the Fe $2p_{3/2}$ peak can be deconvoluted into the Fe^{2+} peak at 709.5~709.8 eV and the Fe^{3+} peak at 710.8~711.1 eV [20]. Comparing the fitting results in Figures S13(a) and (b), the average Fe^{2+}/Fe^{3+} ratio is higher in the 15 Pa film than in the 19 Pa film, suggesting that the 15 Pa film may contain more oxygen vacancies than the 19 Pa film. This agrees with the fact that a lower oxygen pressure used for film growth can lead to a larger amount of oxygen vacancies.

Besides the amounts of oxygen vacancies, the distributions of oxygen vacancies are also different in these two films. As shown in Figure S13(a), the Fe^{2+}/Fe^{3+} ratios at different depths of the 15 Pa film are almost the same, suggesting that the oxygen vacancies are relatively uniformly distributed throughout the film [Figure S13(c)]. By contrast, Figure S13(b) shows that the Fe^{2+}/Fe^{3+} ratio at the surface is higher than those in the bulk regions for the 19 Pa film. This implies that the oxygen vacancies are preferably distributed near the surface of the 19 Pa film [Figure S13(d)].

(b) Possible origin for the difference in oxygen vacancy distribution

To explain the difference in oxygen vacancy distribution, we conjecture that during the growth of a BFO film, a sufficiently high oxygen pressure on the surface may cause the bulk oxygen vacancies to migrate to the surface (using the oxygen pressure gradient as the driving force) [23]. However, when the oxygen pressure on the surface is relatively low, the bulk oxygen vacancies may still remain in the bulk due to the insufficient oxygen pressure gradient.

(c) Correlation between oxygen vacancies and E_{imp}

For the 15 Pa film, the uniform distribution of oxygen vacancies throughout the film can explain the absence of E_{imp}.

For the 19 Pa film, the preferable distribution of oxygen vacancies near the surface may be the origin for the downward E_{imp}. It is known that the mobility of oxygen vacancies is very low at room temperature [24], which can explain why E_{imp} is considerably stable against electric field and time at room temperature (see Figures S10 and S11). However, at high temperature the oxygen vacancies become more mobile [25], and they may migrate toward the bottom interface to compensate the negative polarization charge when the P_{up} state is established beforehand [6,16]. This can explain why the direction of E_{imp} is reversed after the thermal treatment (see Figure S12).

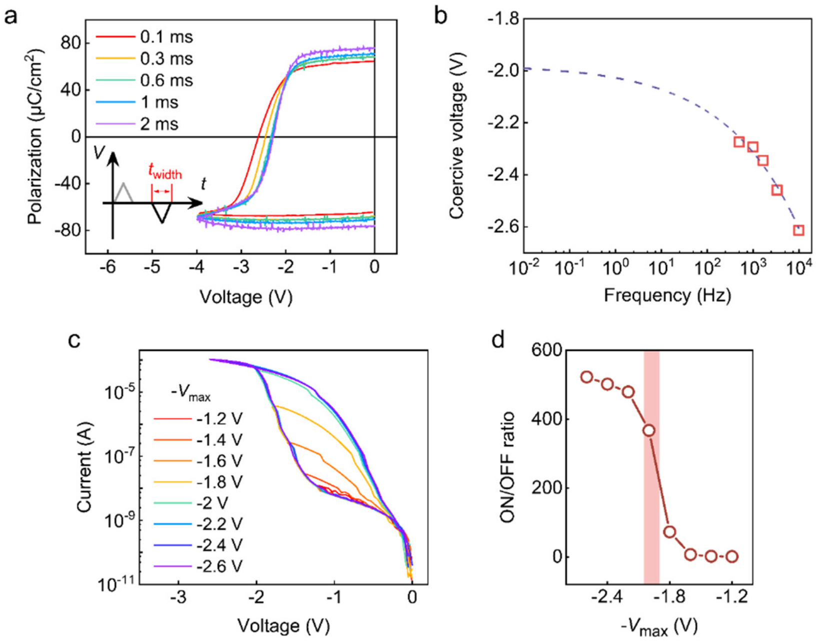

Figure S14. Correlation between polarization switching and resistive switching in the volatile FD. (a) Negative monopolar *P-V* loops at different pulse widths. (b) Negative coercive voltage as a function of frequency (i.e., the reciprocal of pulse width). Hysteretic *I-V* curves measured by applying the voltage sweeps of (c) $0 \rightarrow -V_{max} \rightarrow 0$ ($-V_{max}$ varies from −1.2 V to −2.6 V). (d) ON/OFF ratios (read at −1 V) as a function of $-V_{max}$, as extracted from c. In a and c, the device is set in the initial P_{down} state before each measurement.

Because the volatile FD exhibits one-side diode-type resistive switching behavior where the HRS → LRS switching occurs only in the negative voltage region [Figure 3(c) in the main text], the possible correlation between resistive switching and polarization switching was thus investigated only in the negative voltage region. The method to demonstrate this correlation is by comparing the low-frequency coercive voltage for

polarization switching and the critical voltage for resistive switching, as described in Figure S5.

Figures S14(a) and (b) show that the negative coercive voltage decreases in magnitude as the frequency decreases. By extrapolating the coercive voltage-frequency curve, the negative coercive voltage in the low-frequency region (e.g., 10^{-2} Hz, which is on the same order of the frequency of the *I*-*V* measurement) is roughly estimated as ~−2 V.

On the other hand, as seen from Figures S14(c) and (d), the most abrupt increase in ON/OFF ratio occurs at ~−2 V. This negative critical voltage for resistive switching well corresponds to the negative low-frequency coercive voltage for polarization switching, suggesting that the HRS → LRS switching is induced by the down-to-up polarization switching.

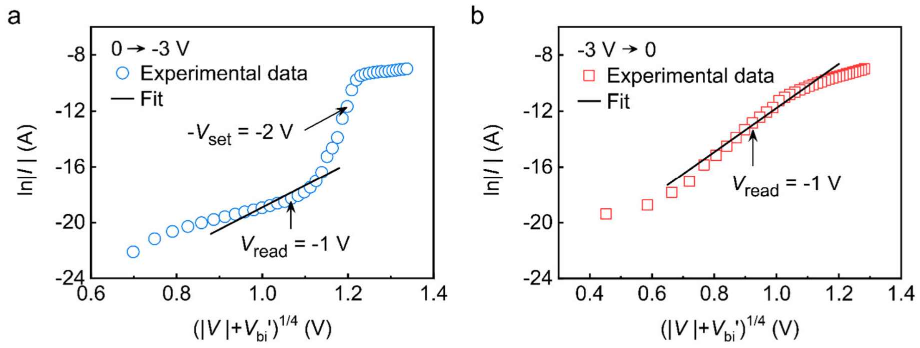

Figure S15. Conduction mechanism analysis for the volatile FD. ln|I| versus $(|V|+V_{bi}')^{1/4}$ curves, as replotted from the I-V curves in Figure 3(c) in the main text, along the voltage sweeps of (a) 0 → −3 V and (b) −3 V → 0. The read voltage and the critical voltage for resistive switching are denoted as V_{read} and $-V_{set}$, respectively, in each panel.

The method for the I-V curve fitting has been described in detail in Figure S6. The ln|I|-$(|V|+V_{bi}')^{1/4}$ curves in both Figures S15(a) and (b) can be well fitted by using the Schottky emission model [Eq. (S1)] in the negative voltage region of interest (i.e., around the read voltage and below the critical voltage for resistive switching). This suggests that for the device in both P_{up} and P_{down} states the Schottky emission may be the dominant conduction mechanism in the negative voltage region.

By using the intercepts of the fitting lines on the ln|I| axis, the heights of the BFO/SRO barrier in the P_{down} and P_{up} states are extracted to be ~1.07 eV and ~0.88 eV, respectively. The barrier height reduction induced by the down-to-up polarization switching is therefore likely to be the origin for the HRS → LRS switching in the negative voltage region.

Note that the I-V curves in the positive voltage region were not used for fitting, because negligible resistive switching occurs in the positive voltage region.

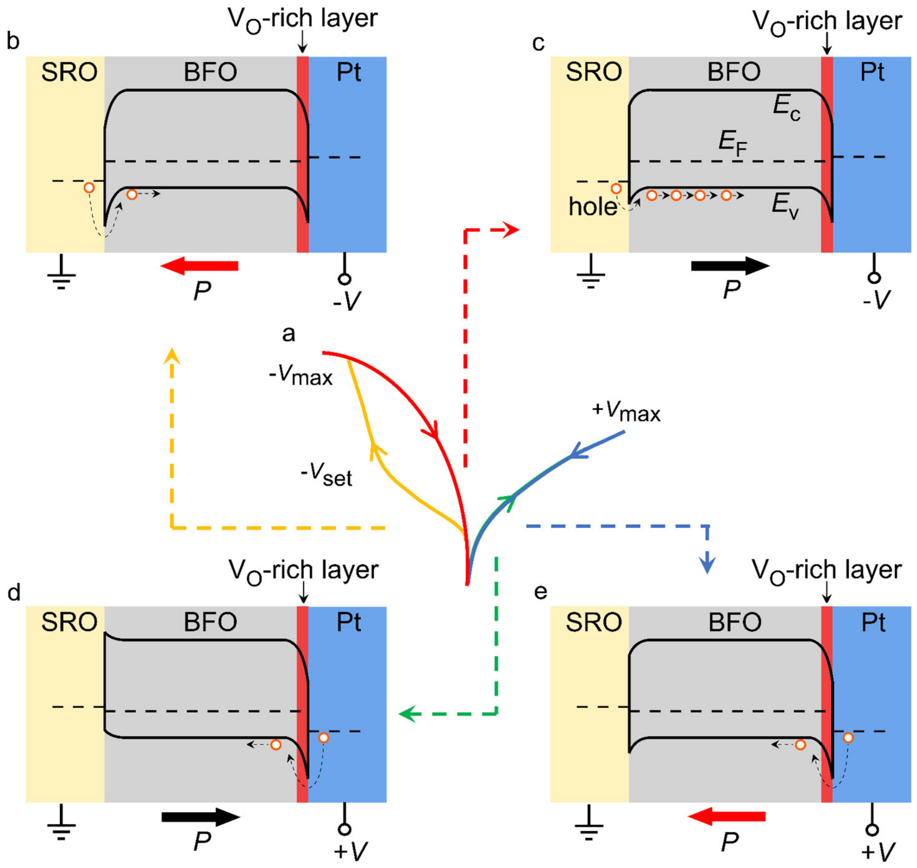

Figure S16. Resistive switching mechanism in the volatile FD. (a) Schematic hysteretic *I*-*V* curves representing the one-side diode-type resistive switching behavior. The negative critical voltage where HRS switches to LRS is indicated by $-V_{\mathrm{set}}$, while the maximum negative and positive applied voltages are indicated by $-V_{\mathrm{max}}$ and $+V_{\mathrm{max}}$, respectively. Schematics showing the energy band diagrams and conduction processes (b) under a small negative voltage in the P_{down} state, (e) under a small negative voltage in the P_{up} state, (f) under a small positive voltage in the P_{up} state, and (g) under a small positive voltage in the P_{down} state.

As the voltage sweeps from 0 to $-V_{\max}$ and back to 0, the BFO/SRO barrier is reverse-biased and thus limits the hole injection. The evolution of the BFO/SRO barrier height has been described in Figure S7. In brief, the BFO/SRO barrier exhibits a relatively large height in the P_{down} state [Figure S16(b)] while its height is reduced upon the down-to-up polarization switching [Figure S16(c)], which in turn causes the HRS → LRS switching.

By contrast, as the voltage sweeps from 0 to $+V_{\max}$ and back to 0, it is the Pt/BFO barrier that is reverse-biased and limits the hole injection. However, as illustrated in Figures S16(d) and (e), the Pt/BFO barrier may be pinned at a high level regardless of the polarization direction due to the oxygen vacancies accumulated near the top interface (Figure S13) [6,16,26]. This may cause the persistence of HRS in the positive voltage region. In addition, some upward polarization already rotates to the downward direction before entering the positive voltage region because of the polarization back-switching, further lowering the driving force for the resistive switching in the positive voltage region.

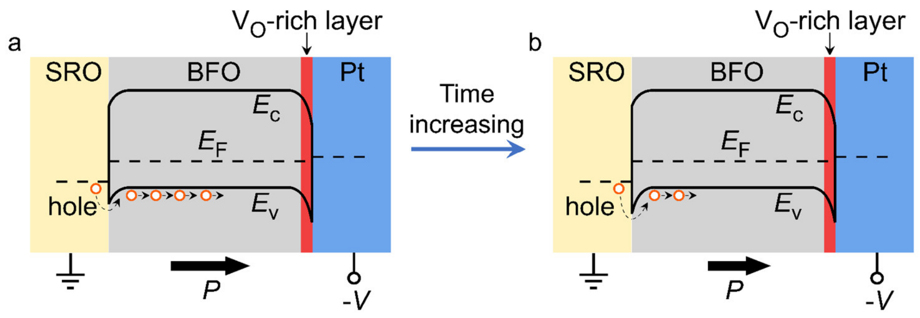

Figure S17. Mechanism of conductance decay in the volatile FD. Schematics showing the energy band diagrams and conduction processes of the device in the P_{up} state (a) before and (b) after polarization back-switching.

As has been demonstrated in Figure S15, the volatile FD exhibits the polarization-controlled Schottky emission in the negative voltage region. In addition, Figure S9 reveals that the upward polarization is unstable and undergoes the back-switching under the effect of the downward E_{imp}. Combing these results, it is deduced that the conductance decay in the P_{up} state [see Figure 3(d) in the main text] may originate from the polarization back-switching that increases barrier height over time. More specifically, when the device is read in the negative voltage region, the BFO/SRO barrier is limiting the conduction. In the initial P_{up} state, the negative polarization charge at the BFO/SRO barrier is large, causing a low barrier height and consequently a high conductance [Figure S17(a)]. However, as time goes by, some upward polarization gets back-switched under the effect of the downward E_{imp}. Therefore, the height of the BFO/SRO barrier gradually increases [Figure S17(b)], leading to the conductance decay.

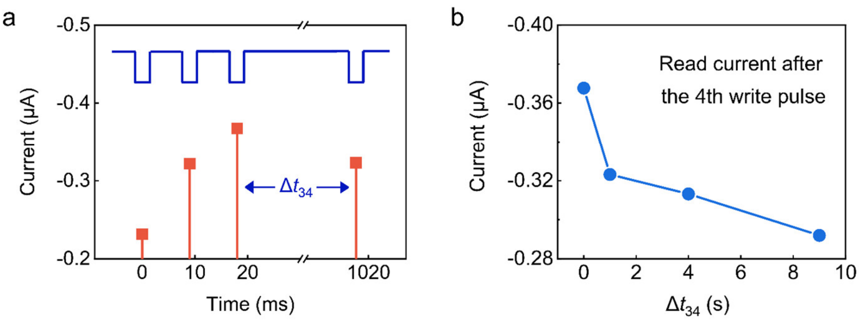

Figure S18. Effect of long interval between write pulses on the conductance of the volatile FD. (a) Current responses (read at –1.2 V) of the volatile FD to multiple write pulses –2.5 V/2 ms), where the interval between the 3rd and 4th write pulses (Δt_{34}) is as long as 1000 ms. (b) The read current after the 4th write pulse as a function of Δt_{34}.

Figure S18(a) shows that the read current gradually increases during the first 3 write pulses due to the short interval (9 ms). However, the read current reduces when the interval between the 3rd and 4th write pulses (Δt_{34}) increases to 1000 ms. Moreover, as seen from Figure S18(b), the longer the Δt_{34} is, the lower the read current becomes.

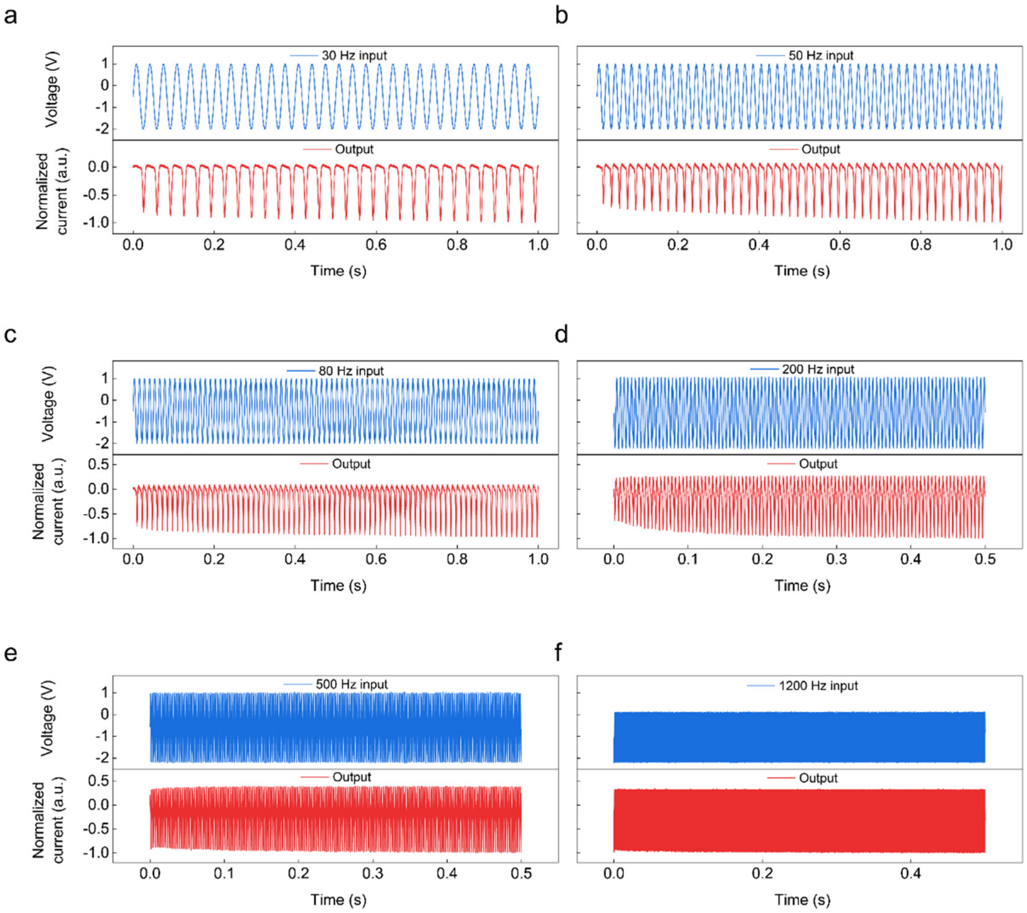

Figure S19. Demonstration of harmonic generation with the volatile FD. Output currents of the volatile FD (lower panel) when stimulated by the sinusoidal voltage waves (upper panel) with frequencies of (a) 30 Hz, (b) 50 Hz, (c) 80 Hz, (d) 200 Hz, (e) 500 Hz, and (f) 1200 Hz.

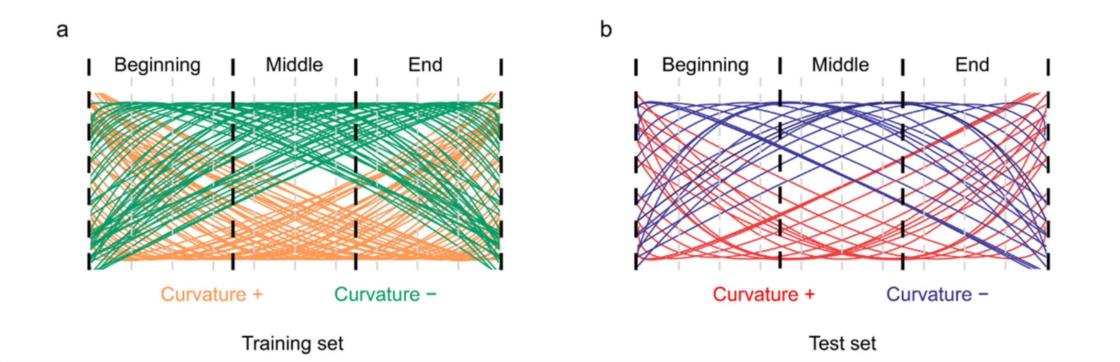

Figure S20. (a) Training set (102 curves) and (b) test set (36 curves) used for the curvature discrimination task.

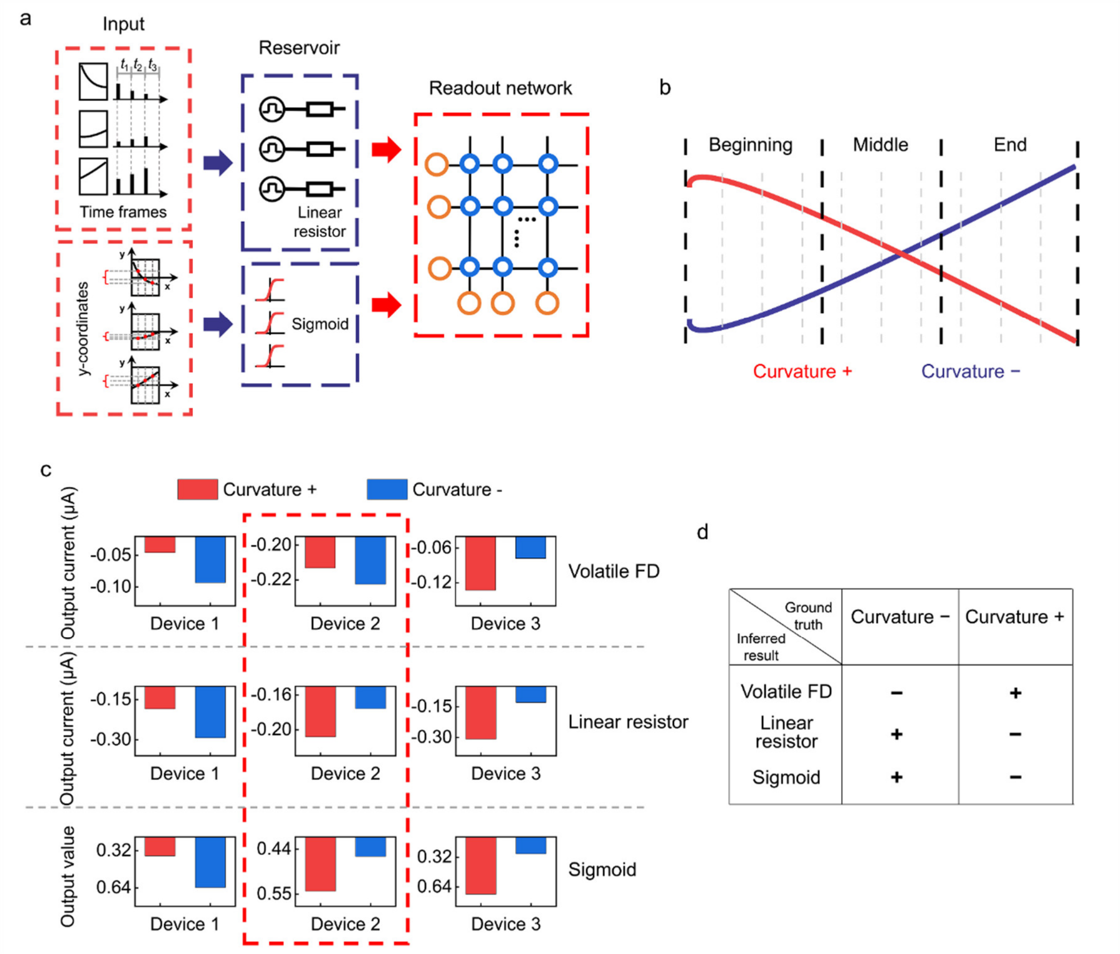

Figure S21. Control experiments of curvature discrimination. (a) Schematics of two control RC systems where the volatile FDs in the reservoir are replaced by linear resistors and sigmoid functions, respectively. (b) Two typical curves from the test set which are misclassified by the two control RC systems. (c) Outputs of the volatile FDs, linear resistors, and sigmoid functions when inputting the curves in b. Device 1-3 correspond to beginning, middle, and end sections of the curve, respectively. (d) Compared results on the discrimination of the curves in b, obtained from the RC systems based on the volatile FDs, linear resistors, and sigmoid functions.

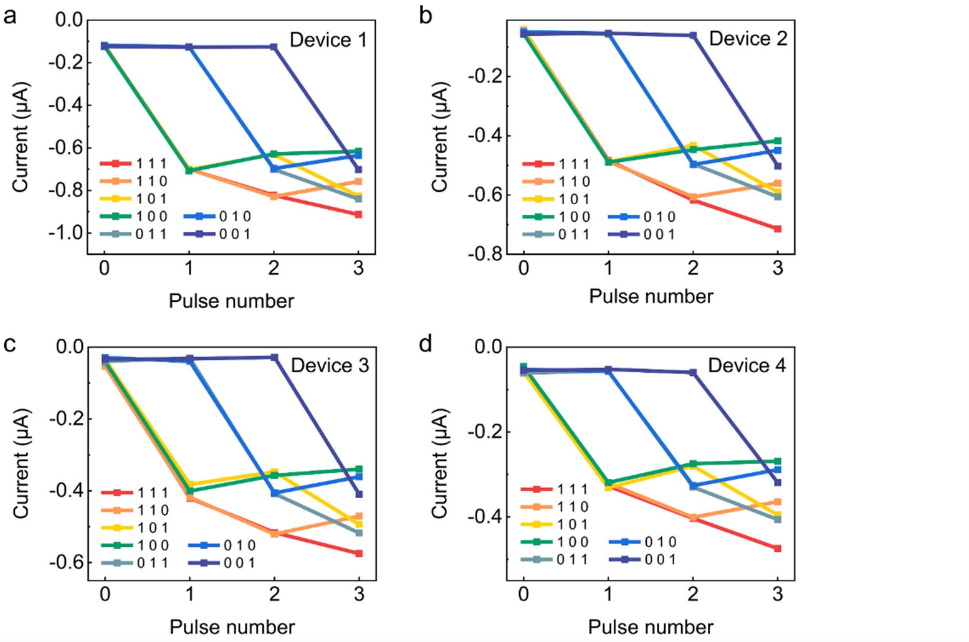

Figure S22. Digit recognition using the volatile FDs as the reservoir. Read current evolutions of (a-d) 4 different volatile FDs under 7 different pulse trains representing the pixel arrangements of "1 1 1", "1 1 0", "1 0 1", "1 0 0", "0 1 1", "0 1 0" and "0 0 1". These 4 volatile FDs together with the one shown in Figure 5 in the main text constitute the reservoir for the digit recognition task.

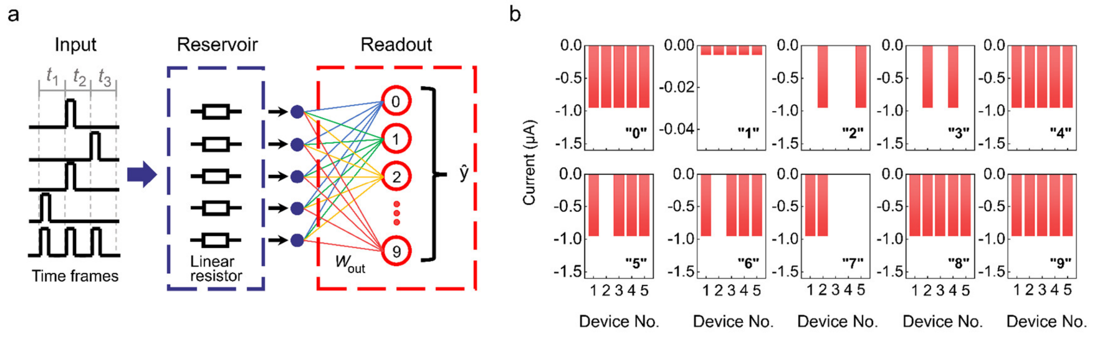

Figure S23. Control experiment of digit recognition. (a) Schematic of a control RC system where the volatile FDs in the reservoir are replaced by linear resistors. (b) Experimentally measured reservoir states after stimulating the linear resistor-based reservoir with different input images in the training set.

We have performed a control experiment of digit recognition, where the volatile FDs in the reservoir were replaced by linear resistors [Figure S23(a)]. The current responses of the linear resistors to write pulses were recorded and no additional read pulses were applied. Since the linear resistor has no memory effect, the last pixel in a row of a digit (i.e., the amplitude of the last pulse in the corresponding pulse train) determines the final current response of the linear resistor. As a result, it is seen from Figure S23(b) that the linear resistors corresponding to different rows produce identical final current responses when the digits “0”, “4”, “8”, and “9” from the training set [see Figure 5(a) in the main text] are presented. This means that the reservoir states corresponding to the digits “0”, “4”, “8”, and “9” are the same, making these digits indistinguishable. Similarly, the digits “5” and “6” from the training set are also indistinguishable. The linear resistor-based reservoir is therefore ineffective.

As a comparison, it is shown in Figure 5(d) in the main text that the volatile FD-based reservoir can produce well distinguishable reservoir states for the 10 digits in the training set. The compared results of Figure S23(b) and Figure 5(d) in the main text therefore demonstrate that the volatile FD plays an essential role in our reservoir.

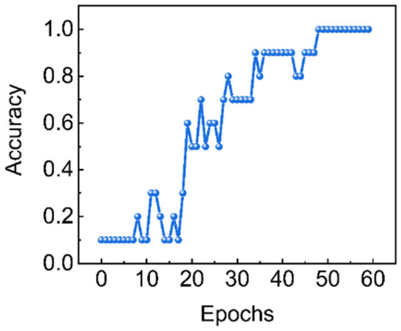

Figure S24. Accuracy evolution of the all-ferroelectric RC system during the training in the digit recognition task.

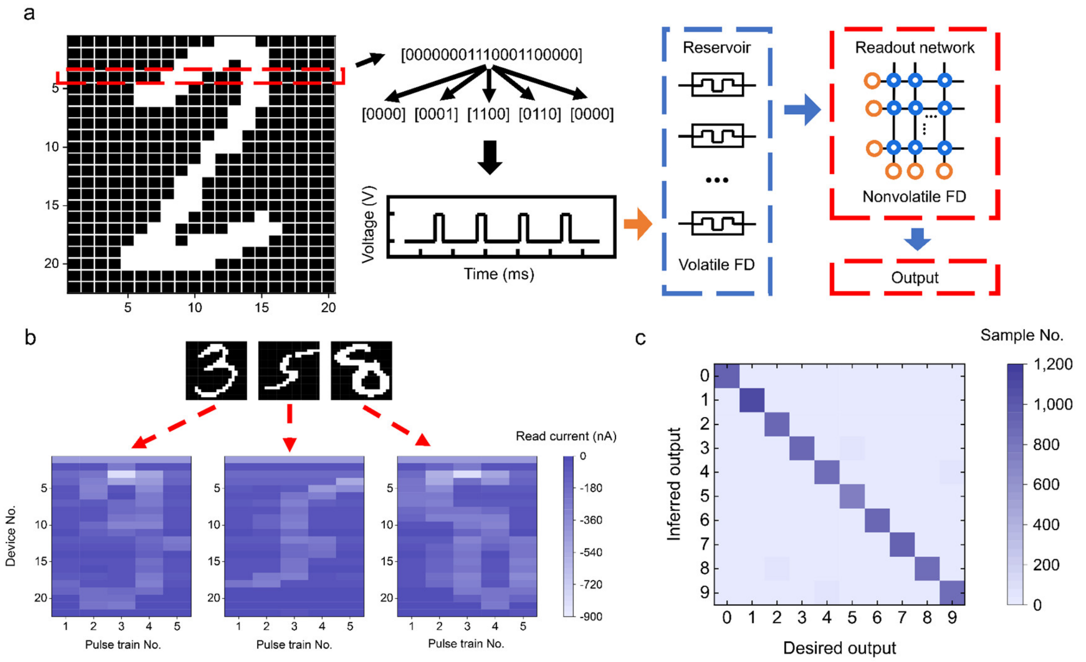

Figure S25. Implementation of MNIST handwritten digit recognition. (a) Schematic flow of the pre-processing of a digit image and the subsequent processing by the all-ferroelectric RC system. (b) Experimentally measured reservoir states corresponding to 3 example digits “3”, “5”, and “8” from the test set. (c) Confusion matrix showing the classification results obtained from the all-ferroelectric RC system versus the target labels.

All the images are pre-processed, as schematically illustrated in Figure S25(a). An original greyscale image is first converted to a binary-pixel image. Then, the unimportant periphery area of the image is removed, reducing the image size from 28 × 28 to 22 × 20. Each row is further chopped into 5 sections, and each section is converted to a 4-timeframe pulse train. The pulse amplitude is −2.7 V (0 V) when the pixel value is 1 (0), and the pulse width is fixed at 2 ms.

After the pre-processing, each image is converted to 22 × 5 pulse trains, which are subsequently fed to a reservoir consisting of 22 volatile FDs. One volatile FD is responsible for processing 5 pulse trains. After each pulse train the device's final conductance state is read out and then its conductance is reset to the initial value through a reset pulse. 110 (22 × 5) read current values are thus obtained, the combination of which forms the reservoir state. The reservoir state is then fed to a (110 + 1) × 10 readout network for classification, where the weights are mapped onto the experimentally measured conductance values of nonvolatile FDs by simulation. The readout network is trained offline with softmax regression, and 60000 images from the MNIST dataset are used for training. After training, the recognition accuracy of the RC system is tested with 10000 images which are not included in the training set.

As an illustrative example, Figure S25(b) shows that the reservoir states corresponding to the digits "3", "5", and "8" are distinctly different, evidencing the effectiveness of the volatile FD-based reservoir. Figure S25(c) shows the confusion matrix produced by our all-ferroelectric RC system on the test set. Most of the digits are correctly classified, and the recognition accuracy reaches 89.5%. This accuracy is 6.5% higher than that achieved by a pioneering diffusive memristor-based RC system [10], whose image pre-processing approach, reservoir architecture, and readout network size are similar to ours. Note that several studies reported even higher accuracies. However, they typically used more complex pre-processing approaches [3,8], different reservoir architectures [27], or multilayer readout networks [1], making it unfair to directly compare their accuracies with ours.

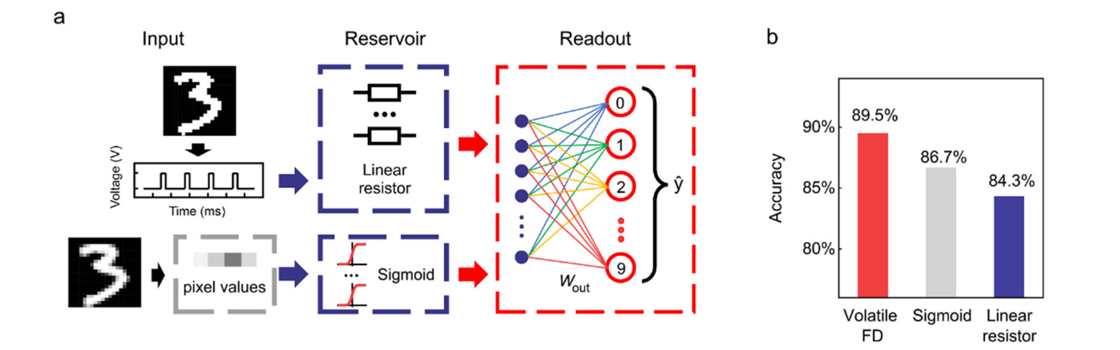

Figure S26. Control experiments of MNIST handwritten digit recognition. (a) Schematics of two control RC systems where the volatile FDs in the reservoir are replaced by linear resistors and sigmoid functions, respectively. (b) Comparison of accuracies on the test set achieved by the volatile FD-, linear resistor-, and sigmoid-based RC systems.

It is seen that the linear resistor-based RC system achieves 84.3% accuracy on the test set, while the sigmoid-based RC system achieves a higher accuracy of 86.7%. The higher performance of the sigmoid-based RC system may be attributed to the nonlinearity of the sigmoid function. However, the accuracies of both the two control systems are lower than the accuracy of 89.5% achieved by the volatile FD-based RC system (i.e., the all-ferroelectric RC system). It is deducible that the combined memory effect and nonlinearity of the volatile FD lead to the high performance of the volatile FD-based RC system.

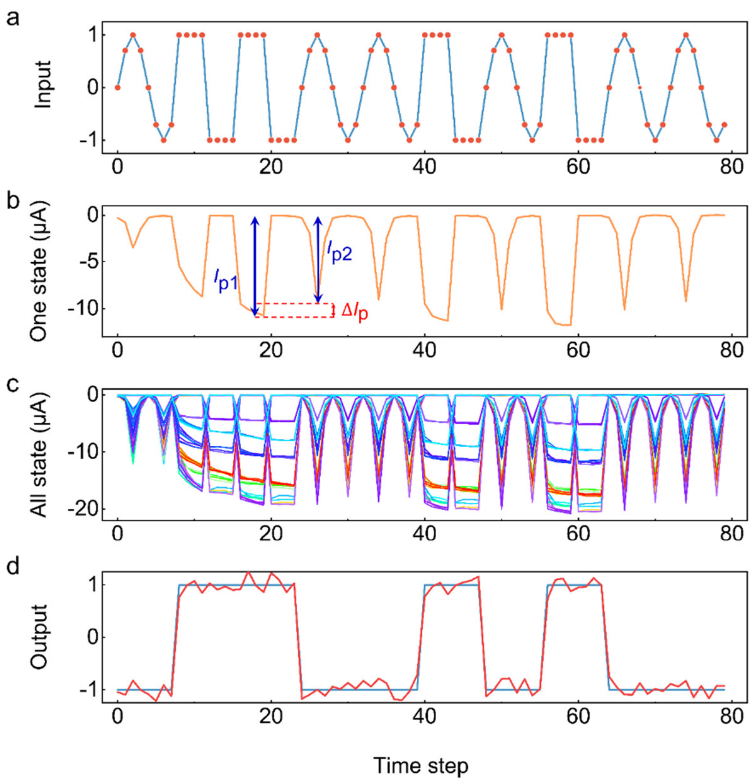

Figure S27. Waveform classification using the volatile FDs as the reservoir. (a) Input waveform, which is transformed into the pulse trains through a mask process. (b) A typical virtual-node state and (c) all virtual-node states of the 8-volatile-FD reservoir. (d) Classification results obtained from the all-ferroelectric RC system.

The feedback strength can be represented by the difference between the peaks of the volatile FD's responses to the input square and sine waveforms (I_{p1} and I_{p2}, respectively), while the state richness can be reflected by the deviation of the I_{p2} values produced by different virtual nodes. As seen from Figures S27(b) and (c), both the feedback strength and the state richness are relatively high, which are the keys to obtain good classification results.

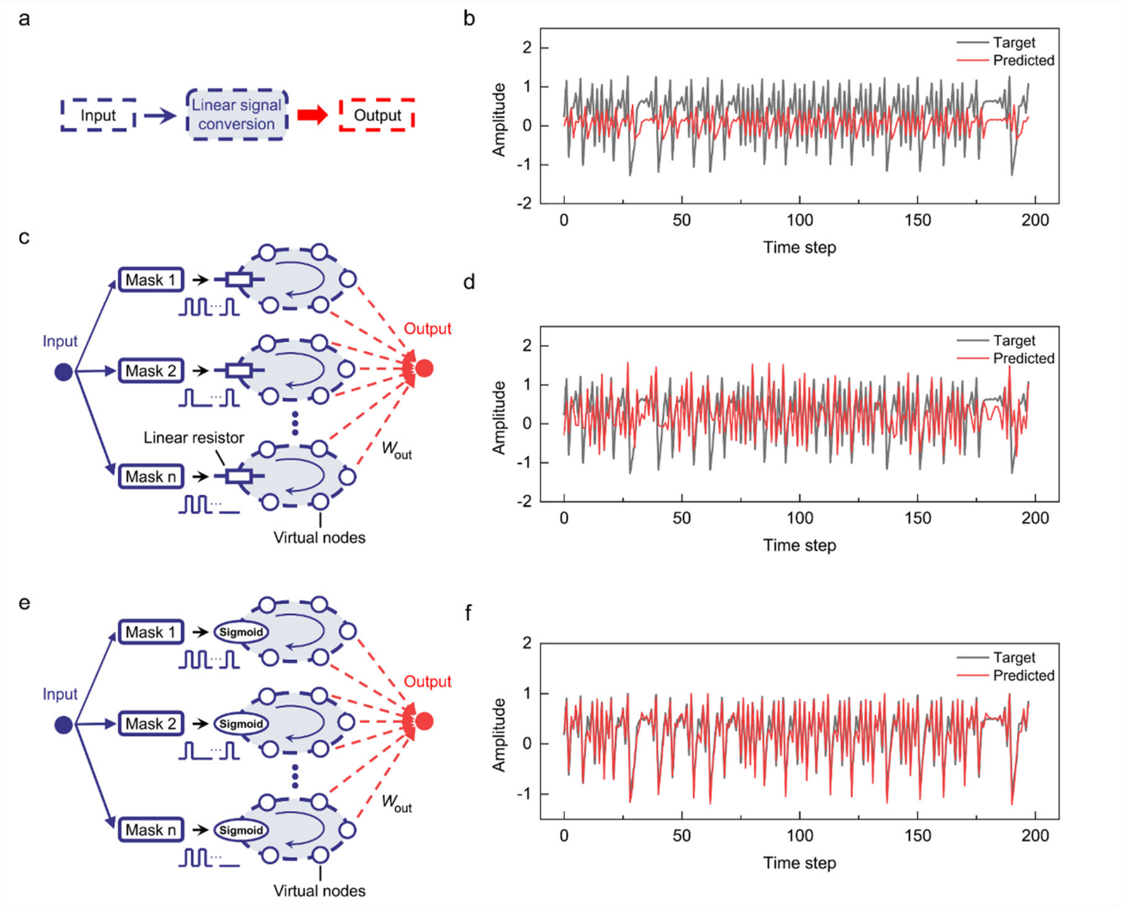

Figure S28. Control experiments of Hénon map prediction. (a) Schematic of the first control RC system where the reservoir and mask process are removed. (c) Schematic of the second control RC system where the volatile FDs in the reservoir are replaced by linear resistors. (e) Schematic of the third control RC system where the volatile FDs in the reservoir are replaced by sigmoid functions. (b,d,f) Predicted time series versus ideal targets obtained from the RC systems shown in a, c, and e, respectively.

The results shown in Figures S28(a) and (b) indicate that the pre-processing and post-processing alone cannot result in good RC performance. The results in Figures S28(c)-(f) demonstrate that the volatile FD-based reservoir is essential to the RC performance. Detailed analyses can be found in Supplementary Note 3.

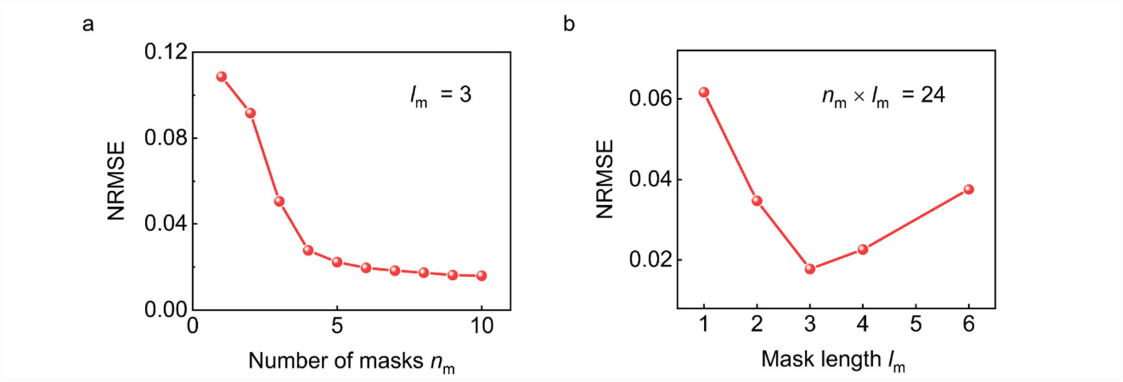

Figure S29. Effect of mask process on the Hénon map prediction performance. NRMSE on the test set as a function of (a) number of masks n_m (mask length l_m fixed at 3), and (b) mask length l_m ($l_m \times n_m$ fixed at 24).

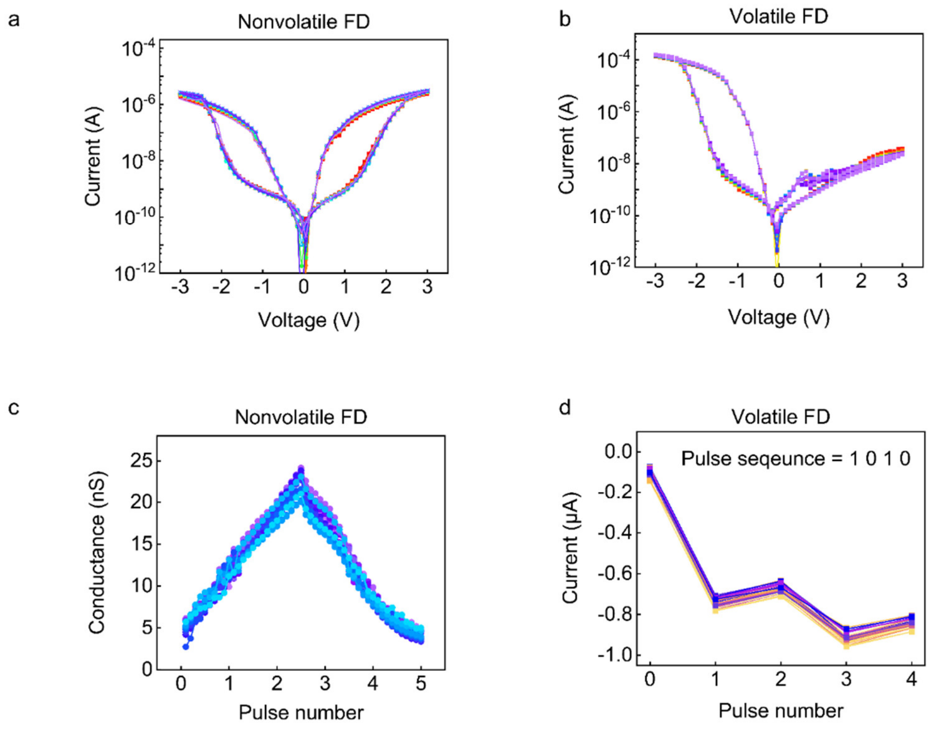

Figure S30. C2C variations. Multi-cycle *I*-*V* characteristics (15 cycles) for (a) nonvolatile and (b) volatile FDs. (c) Condensed plot of the LTP/LTD processes (15 cycles) of the nonvolatile FD. (d) Read current evolutions of the volatile FD under 30 repeated "1 0 1 0" pulse trains, where "1" represents a –2.5 V/2 ms pulse while "0" represents a 0 V pulse.

Figures S30(a) and (b) show the multi-cycle *I*-*V* curves of the nonvolatile and volatile FDs, respectively, as measured by the cyclic DC voltage sweeps. For both types of devices, the *I*-*V* curves in different cycles almost overlap with each other. Based on these results, the C2C variations (characterized by the DC voltage measurements) of the nonvolatile and volatile FDs are calculated to be ~6% and ~7%, respectively.

Figure S30(c) shows the multi-cycle LTP/LTD characteristics of the nonvolatile FD. It is seen that the LTP/LTD curves in different cycles largely overlap with each other,

demonstrating that the multilevel conductance states are repeatable during the cyclic LTP/LTD pulse measurements. On the other hand, Figure S30(d) displays the read current evolutions of the volatile FD under 30 repeated "1 0 1 0" pulse trains. The read current responses in different cycles show little deviations. Based on these results, the C2C variations (characterized by the pulse measurements) of the nonvolatile and volatile FDs are calculated to be ~8% and ~6%, respectively.

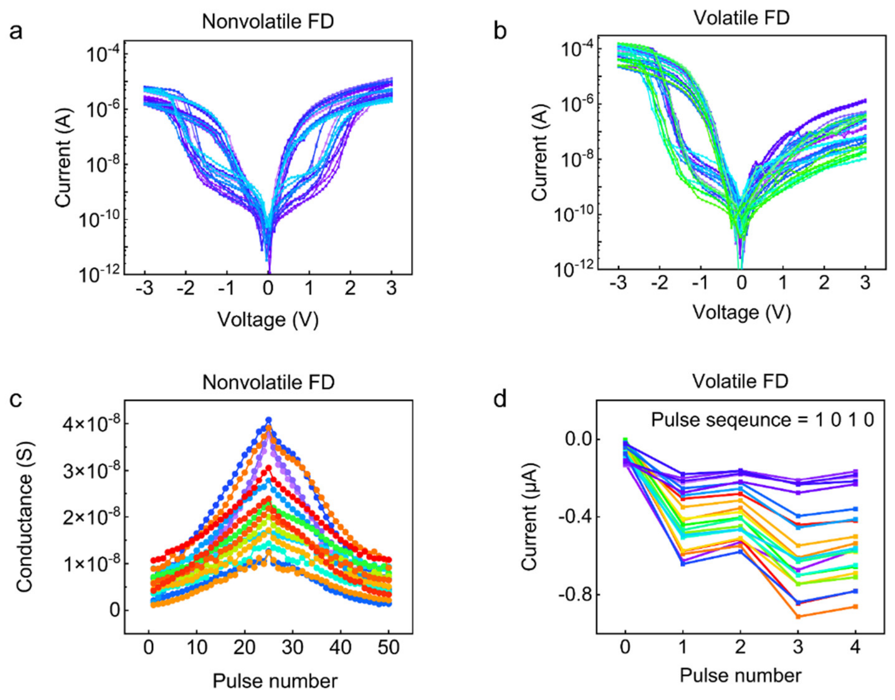

Figure S31. D2D variations. *I-V* characteristics for (a) 15 nonvolatile FDs and (b) 22 volatile FDs. (c) LTP/LTD processes measured from 15 nonvolatile FDs. (d) Read current evolutions of 22 volatile FDs under the same pulse train of "1 0 1 0", where "1" represents a –2.5 V/2 ms pulse while "0" represents a 0 V pulse.

Figures S31(a) and (b) present the *I-V* curves for multiple devices of nonvolatile and volatile FDs, respectively. The *I-V* curves deviate rather greatly from each other. The D2D variations (characterized by the DC voltage measurements) of the nonvolatile and volatile FDs are calculated to be ~56% and ~60%, respectively.

Figure S31(c) shows the LTP/LTD processes measured from 15 nonvolatile FDs. The LTP/LTD curves from different devices have relatively wide distributions. On the other hand, Figure S31(d) presents the read current evolutions of 22 volatile FDs under the same "1 0 1 0" pulse train. The read current responses from different devices show relatively

large deviations. Based on these results, the D2D variations (characterized by the pulse measurements) of the nonvolatile and volatile FDs are calculated to be ~40% and ~45%, respectively.

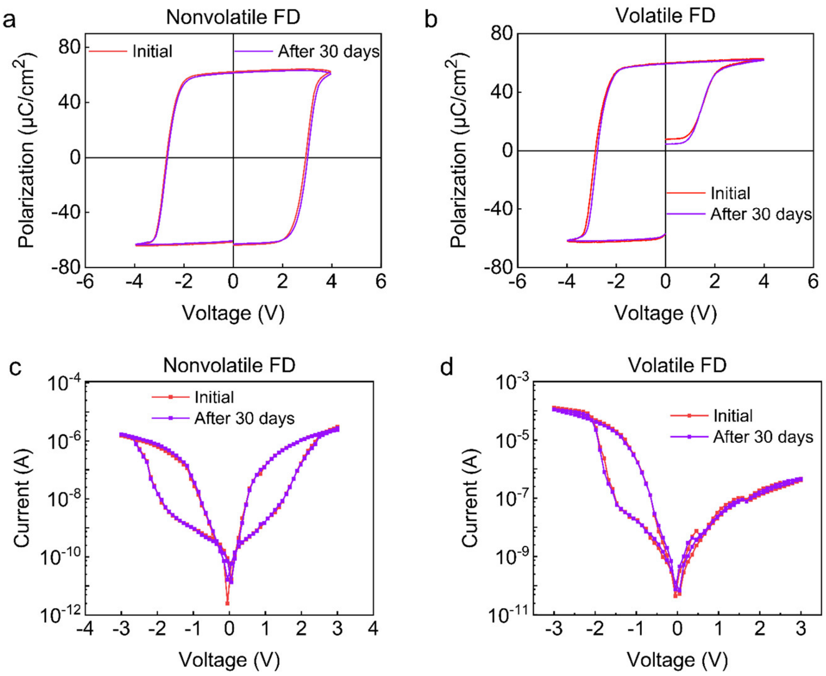

Figure S32. Long-term stability in the ambient air. *P-V* loops of (a) nonvolatile and (b) volatile FDs before and after a 30-day exposure to the ambient air. *I-V* characteristics of (c) nonvolatile and (d) volatile FDs before and after a 30-day exposure to the ambient air.

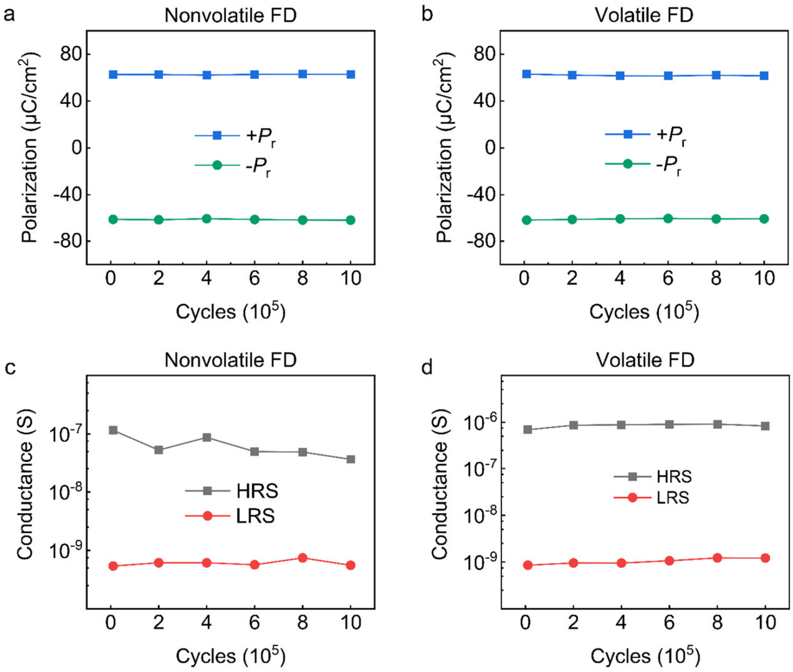

Figure S33. Endurance. Polarizations as a function of switching cycle for (a) nonvolatile and (b) volatile FDs. Conductances in HRS and LRS as a function of switching cycle for (c) nonvolatile and (d) volatile FDs. The pulses used to switch the devices are ±3 V/5 μs. In b, the $-P_r$ values of the volatile FD are those before relaxation.

Table S2. Parameters of the curves used in the curvature discrimination task. The first 102 curves constitute the training set, while the test set is composed of the rest 36 curves.

Curve number	a	θ (degree)	p	q
1	0.8	-30	3.6	3.45
2	0.8	-30	3.6	3.5
3	2.5	-42	4	3.5
4	1	-30	3.6	3.5
5	0.5	-20	3.2	3.5
6	0.6	-20	3.1	3.5
7	0.45	-0.5	0	3.55
8	0.4	-0.5	0	3.55
9	0.4	-5	0.6	3.55
10	0.4	-5	0.8	3.55
11	0.3	-5	0.5	3.55
12	0.5	-20	3	3.4
13	0.4	-22	3.1	3.4
14	0.45	-22	2.9	3.4
15	0.4	-16	2.5	3.5
16	0.35	-16	2.5	3.5
17	0.35	-12	2.2	3.5
18	0.3	-14	2	3.5
19	0.4	-5	1.2	3.5
20	0.35	-5	1.2	3.5
21	0.35	-5	1.6	3.5
22	0.35	-10	1.6	3.5
23	0.28	-13	1.6	3.5
24	0.28	13	-1.6	3.5
25	0.35	10	-1.6	3.5
26	0.35	5	-1.6	3.5
27	0.35	5	-1.2	3.5
28	0.4	5	-1.2	3.5
29	0.3	14	-2	3.5
30	0.35	12	-2.2	3.5
31	0.35	16	-2.5	3.5
32	0.4	16	-2.5	3.5
33	0.45	22	-2.9	3.4
34	0.4	22	-3.1	3.4
35	0.5	20	-3	3.4
36	0.3	5	-0.5	3.55
37	0.4	5	-0.8	3.55
38	0.4	5	-0.6	3.55
39	0.4	0.5	0	3.55
40	0.45	0.5	0	3.55
41	0.6	20	-3.1	3.5
42	0.5	20	-3.2	3.5
43	1	30	-3.6	3.5
44	1	30	-3.8	3.5
45	1.3	30	-3.8	3.5
46	2.5	42	-4	3.5
47	2.5	38	-4	3.5

48	3.2	36	-3.9	3.5
49	3	36	-3.9	3.5
50	0.8	30	-3.6	3.5
51	0.8	30	-3.6	3.45
52	0.8	-150	3.6	-3.45
53	0.8	-150	3.6	-3.5
54	2.5	-138	4	-3.5
55	1	-150	3.6	-3.5
56	0.5	-160	3.2	-3.5
57	0.6	-160	3.1	-3.5
58	0.45	-179.5	0	-3.55
59	0.4	-179.5	0	-3.55
60	0.4	-175	0.6	-3.55
61	0.4	-175	0.8	-3.55
62	0.3	-175	0.5	-3.55
63	0.5	-160	3	-3.4
64	0.4	-158	3.1	-3.4
65	0.45	-158	2.9	-3.4
66	0.4	-164	2.5	-3.5
67	0.35	-164	2.5	-3.5
68	0.35	-168	2.2	-3.5
69	0.3	-166	2	-3.5
70	0.4	-175	1.2	-3.5
71	0.35	-175	1.2	-3.5
72	0.35	-175	1.6	-3.5
73	0.35	-170	1.6	-3.5
74	0.28	-167	1.6	-3.5
75	0.28	167	-1.6	-3.5
76	0.35	170	-1.6	-3.5
77	0.35	175	-1.6	-3.5
78	0.35	175	-1.2	-3.5
79	0.4	175	-1.2	-3.5
80	0.3	166	-2	-3.5
81	0.35	168	-2.2	-3.5
82	0.35	164	-2.5	-3.5
83	0.4	164	-2.5	-3.5
84	0.45	158	-2.9	-3.4
85	0.4	158	-3.1	-3.4
86	0.5	160	-3	-3.4
87	0.3	175	-0.5	-3.55
88	0.4	175	-0.8	-3.55
89	0.4	175	-0.6	-3.55
90	0.4	179.5	0	-3.55
91	0.45	179.5	0	-3.55
92	0.6	160	-3.1	-3.5
93	0.5	160	-3.2	-3.5
94	1	150	-3.6	-3.5
95	1	150	-3.8	-3.5
96	1.3	150	-3.8	-3.5
97	2.5	138	-4	-3.5
98	2.5	142	-4	-3.5
99	3.2	144	-3.9	-3.5
100	3	144	-3.9	-3.5

101	0.8	150	-3.6	-3.5
102	0.8	150	-3.6	-3.45
103	1	-30	3.6	3.45
104	3	-36	3.9	3.5
105	3.2	-36	3.9	3.5
106	0.4	-5	1.1	3.55
107	0.45	-20	2.9	3.4
108	0.35	-14	2.2	3.5
109	0.4	-10	1.2	3.5
110	0.35	-10	1.2	3.5
111	0.26	-10	1.4	3.55
112	1	30	-3.6	3.45
113	0.4	5	-1.1	3.55
114	0.45	20	-2.9	3.4
115	0.35	14	-2.2	3.5
116	0.4	10	-1.2	3.5
117	0.35	10	-1.2	3.5
118	0.26	10	-1.4	3.55
119	0.42	0	0	3.55
120	2.5	-34	3.87	3.55
121	1	-150	3.6	-3.45
122	3	-144	3.9	-3.5
123	3.2	-144	3.9	-3.5
124	0.4	-175	1.1	-3.55
125	0.45	-160	2.9	-3.4
126	0.35	-166	2.2	-3.5
127	0.4	-170	1.2	-3.5
128	0.35	-170	1.2	-3.5
129	0.26	-170	1.4	-3.55
130	1	150	-3.6	-3.45
131	0.4	175	-1.1	-3.55
132	0.45	160	-2.9	-3.4
133	0.35	166	-2.2	-3.5
134	0.4	170	-1.2	-3.5
135	0.35	170	-1.2	-3.5
136	0.26	170	-1.4	-3.55
137	0.42	-180	0	-3.55
138	2.5	-146	3.87	-3.55

Supplementary Note 1. Dataset for the curvature discrimination task

In the curvature discrimination task, the original curve follows the equation:

$$y = ax^2 \tag{S2}$$

where a is a parameter which can be varied. The curve is then rotated around the origin by an angle of θ (θ is a variable parameter). Afterward, the curve is shifted horizontally and vertically by p and q, respective, where p and q are also variable parameters. The horizontal coordinate interval of the curve is set to be [-4, 4]. By varying the parameters a, θ, p, and q (see Table S2), 138 different curves are generated. 102 curves are then selected from the whole 138 curves and constitute the training set [see Figure S20(a)], while the rest 36 curves constitute the test set [see Figure S20(b)].

Supplementary Note 2. Possible factors influencing the performance of curvature discrimination

In the curvature discrimination experiment, the pre-processing mainly includes 2 steps: 1) chopping each curve into 3 sections, and 2) converting each section to a 3-timeframe pulse train. The beginning, middle, and end sections of a curve are therefore represented by 3 pulse trains, respectively. The 3 pulse trains are then applied to a reservoir consisting of 3 volatile FDs, with each device processing one pulse train.

As seen above, the key function of the pre-processing is converting the spatial information in the curve into the temporal features in the streaming inputs. How the curve is pre-processed thus affects the temporal features to be extracted by the volatile FDs, which in turn influences the classification accuracy.

For example, if the curve is not chopped, one 9-timeframe pulse train is sufficient to represent this curve (assuming that the product of the number of pulse trains and that of timeframes is 9). Correspondingly, only one volatile FD is used to process this pulse train. Due to the short-term memory of the volatile FD, only the spatial information near the end of the curve can be well captured while that in the beginning and middle may be lost, resulting in a poor accuracy. On the other hand, if the curve is chopped into too many sections, e.g., 9 sections, 9 1-timeframe pulse trains are needed to represent this curve (also assuming that the product of the number of pulse trains and that of timeframes is 9). Correspondingly, 9 volatile FDs are used to process the 9 pulse trains. Because each pulse train has only 1 timeframe, it is unable to use the memory effect of the volatile FDs. The reservoir in this case is ineffective.

Therefore, to take full advantage of the volatile FD-based reservoir, we have chopped the curve into an appropriate number of sections (i.e., 3 sections), and converted each section to a pulse train with an appropriate number of timeframes (i.e., 3 timeframes). Similar way of pre-processing has been widely used for physical RC systems when handling spatial pattern recognition tasks [27,28].

Although the pre-processing has certain effects on the RC performance, we argue that it plays an auxiliary role. The volatile FD-based reservoir indeed plays the essential role because it is responsible for extracting temporal features. This can be evidenced through control experiments as follows.

In the control experiments, the volatile FDs in the reservoir were replaced by linear resistors and sigmoid functions [Figure S21(a)], while the approaches of curve chopping, linear regression, and nonlinear post-processing (sigmoid activation function) were kept the same as those for the volatile FD-based RC system. For the linear resistor-based RC system, the current responses of the linear resistors to write pulses were recorded and directly used for reservoir states, while no additional read pulses were applied. For the sigmoid-based RC system, each section of an input curve was directly processed by a sigmoid function. The sigmoid function is a simple nonlinear function without memory effect, which is expressed by

$$f(x) = \frac{1}{1+e^{-x-t}}, \tag{S3}$$

where the input x is the y-coordinate of the point on the input curve, and t is a parameter which can be optimized. Note that for different tasks, x and t can be changed accordingly.

As shown in Figure 4f in the main text, both the linear resistor- and sigmoid-based RC systems achieve the same accuracy of 83.3% on the test set. The same accuracy of the two control systems may be caused by the fact that both the linear resistor and sigmoid function have no memory effect. In addition, the same accuracy in turn suggests that the nonlinearity of the sigmoid function does not contribute to the RC performance in this simple task.

Figure 4f in the main text also presents that the accuracies of the two control systems are apparently lower than the 100% accuracy of the volatile FD-based RC system (i.e., the all-ferroelectric RC system). To understand why the all-ferroelectric RC system outperforms the two control systems, typical misclassified results obtained from the two control systems are specifically analyzed. Figure S21(b) displays two typical curves from the test set which are misclassified by the two control systems. When inputting the curve with negative (positive) curvature, both the linear resistor and sigmoid function corresponding to the middle section of the curve produce a lower (higher) output, as shown in Figure S21(c). This is the main cause for the two control systems to make wrong classification [Figure S21(d)] given the readout weights trained offline. Why the linear resistor and sigmoid function produce such outputs is simply because they have no memory effect and hence the relative height of the last point in the middle section of the curve determines the magnitude of the output.

By contrast, the volatile FD produces different current outputs [see Figure S21(c)], which is well attributed to its memory effect allowing the pulse history to influence the conductance. This in turn results in a correct classification given the readout weights trained offline, as shown in Figure S21(d).

The above compared results therefore confirm that the volatile FD-based reservoir is the key for the superior performance of our RC system in the curvature discrimination. Besides, the nonvolatile FDs, which provide multilevel nonvolatile conductance states for the mapping of readout weights, are also important for the RC performance.

Supplementary Note 3. Possible factors contributing to the high performance in the Hénon map prediction

In the experiment of Hénon map prediction, there are several possible factors which may influence the performance, such as the volatile FD-based reservoir, nonvolatile FD-based readout network, pre-processing, and post-processing. These factors are analyzed in detail as follows.

(a) Role of post-processing

The readout network was trained with linear regression in this experiment. No nonlinear post-processing was performed. There is thus little contribution from the post-processing to the RC performance, which can be evidenced by a control experiment presented in Section (b).

(b) Role of pre-processing (linear signal conversion)

The pre-processing mainly involves two processes: 1) the mask process which can generate virtual nodes, and 2) the linear conversion of input signals to pulse voltages. The role of the mask process will be discussed later. The linear conversion of input signals to pulse voltages is a widely used way to pre-process the time-series data [7,8], and such linear signal conversion may contribute little to the RC performance [29].

To confirm the minor roles played by the pre-processing of linear signal conversion and the post-processing of linear regression, we have performed a control experiment. The input signals $x(n)$ and $x(n-1)$, after the linear signal conversion, were directly fed to a readout network which was trained by the linear regression. Neither mask process nor reservoir was used. The predicted time series are shown in Figure S28(b), which deviate

significantly from their corresponding ideal targets. The NRMSE value on the test set is further calculated to be 0.98, which is extremely high. These results demonstrate that the pre-processing of linear signal conversion and the post-processing of linear regression are minor factors contributing to the RC performance.

(c) Role of mask process

The mask process is known to be capable of improving the RC performance since it can generate virtual nodes to effectively expand the reservoir size [7,8]. To demonstrate this in our RC system, the RC performance was investigated with varying number of masks (n_m) and mask length (l_m). In the experiment of Hénon map prediction, each mask sequence is processed by one volatile FD, generating l_m virtual nodes. The reservoir size, or the total number of virtual nodes, is thus $l_m \times n_m$.

Figure S29(a) shows that NRMSE decreases with increasing n_m (l_m fixed at 3). This is within expectation because the reservoir size becomes larger when using more masks. More reservoir states are thus generated, which can help to better capture the features of the input signals. In addition, as more masks are used, more volatile FDs are also used correspondingly. The device-to-device variation of the volatile FDs can help to expand the effective reservoir size, which is an additional factor contributing to the decrease of NRMSE.

Then, the effect of mask length l_m was investigated by varying l_m while fixing $l_m \times n_m$ at 24. Figure S29(b) shows that NRMSE first decreases and then increases with increasing l_m, and it reaches the minimum value of 0.017 at $l_m = 3$. When the mask length is too short, e.g., $l_m = 1$, the number of different types of mask sequences is very small. Hence, the

richness of reservoir states is very low, causing a high NRMSE. Increasing l_m can improve the reservoir state richness, thus reducing NRMSE. However, as l_m further increases, the decrease of feedback strength becomes dominant [8]. This is the main cause for the rise of NRMSE with increasing M. In addition, the role of the device-to-device variation becomes weaker as l_m increases (given that $l_m \times n_m$ is fixed at 24, a larger l_m leads to a smaller n_m). This is an additional factor contributing to the rise of NRMSE.

As Figure S29(b) shows that $l_m = 3$ is the optimal mask length under the constraint of $l_m \times n_m = 24$, we therefore report the results obtained at $l_m = 3$ and $n_m = 8$ in the main text. However, it should be noted that the optimal value of l_m is task-dependent, and it may change if different pulse parameters and different constraints of $l_m \times n_m$ are used.

Although the above results have shown that the mask process contributes to the RC performance, it indeed plays an auxiliary role. The decisive factors are actually the device characteristics of the volatile and nonvolatile FDs, as demonstrated as follows.

(d) Roles of device characteristics of volatile and nonvolatile FDs

For an RC system, the nonlinearity and short-term memory of the reservoir and the multilevel nonvolatile weights of the readout network are of critical importance to the performance. These functional requirements of the reservoir and readout network are well fulfilled by our volatile and nonvolatile FDs, respectively.

For the nonvolatile FD, it is engineered to be free from E_{imp}, so it can exhibit good polarization stability and consequent nonvolatile memristive switching. The multilevel nonvolatile conductance states (>4 bits) can be used to precisely map the weights in the readout network, which is apparently critical for the RC performance.

On the other hand, the volatile FD with purposely introduced E_{imp} is used to implement the reservoir. Thanks to the complex polarization dynamics (including the nonlinear, history-dependent polarization switching under external field and spontaneous polarization back-switching induced by E_{imp}), the volatile FD well exhibits the nonlinearity and short-term memory as required by a reservoir. To demonstrate that the volatile FD-based reservoir is essential to the RC performance, we replaced the volatile FDs in the reservoir with linear resistors and sigmoid functions (without changing other factors like the mask process and the readout network) and investigated how the performance would change. As shown in Figure S28(c)-(f), the control RC systems using linear resistor- and sigmoid-based reservoirs yield rather poor predicted results in the Hénon map prediction task. Their NRMSE values on the test set are as high as 0.70 and 0.24, respectively (the lower NRMSE value of the sigmoid-based control system may be attributed to the nonlinearity of the sigmoid function). Nevertheless, the two control systems' NRMSE values are much higher than the value of 0.017 achieved by the all-ferroelectric RC system using a volatile FD-based reservoir. This unambiguously demonstrates that the volatile FD-based reservoir is the key to the RC performance.

Note that in the control RC systems using linear resistor- and sigmoid-based reservoirs, although the mask process (l_m = 3 and n_m = 8) is used, the performance is still poor. This is because the linear resistor and sigmoid function have no memory effect, causing the virtual nodes generated by the mask process to be independent of each other. Hence, the linear resistor- and sigmoid-based reservoirs are unable to capture the features in the temporal inputs, even though the mask process is used. This in turn suggests that the mask process alone could not result in good RC performance.

In fact, the mask process plays an auxiliary role, i.e., expanding the reservoir size. It works only under the condition that the devices in the reservoir (like our volatile FDs) possess nonlinearity and short-term memory. With such device characteristics, the virtual nodes are nonlinearly coupled, and the current state of a virtual node depends on its own previous state, the current state of its neighboring nodes, and the input signal applied to this node. The reservoir is hence capable of capturing both local temporal features within an input window and more global features among input windows, leading to good RC performance.

(e) Physical mechanisms underlying the device characteristics

As demonstrated above, the good performance of our all-ferroelectric RC system in the Hénon map prediction can be attributed to the following factors: the device characteristics of the volatile and nonvolatile FDs (essential) and the mask process (auxiliary). Below we will further analyze the physical mechanisms underlying the device characteristics, so as to gain deeper insights into why the performance is so good.

As shown in Eqs. (1) and (2) in the main text, the Hénon map prediction is a nonlinear 2D mapping problem, where each output depends on the recent past results but not on the far past. Such problem is well suited for reservoirs based on memristors with nonlinearity and short-term memory, like our volatile FDs.

The nonlinearity of our volatile FD mainly originates from both the nonlinear polarization switching and the nonlinear polarization-controlled conduction behavior. The polarization switching typically involves two microscopic processes: domain nucleation and domain growth, both of which have strong nonlinear dependencies on the applied

voltage [2]. Additionally, the domains in the volatile FD are observed to be tiny and irregularly-shaped (Figure S3), which typically results in a wide distribution of switching voltages. This could allow the nonlinear polarization switching to occur in a relatively wide range of voltages, covering the voltages applied in the Hénon map prediction task (the applied pulse voltages are within –2 V, and –2 V is around the coercive voltage where strong nonlinearity exists). Besides the nonlinear polarization switching, the nonlinear polarization-controlled conduction behavior, where the current is nonlinearly dependent on the polarization-controlled Schottky barrier height and the applied voltage [30], further adds to the device's nonlinearity.

In terms of the short-term memory in our volatile FD, it mainly originates from both the history dependence of polarization switching and the spontaneous polarization back-switching induced by E_{imp}. Some previously reported ferroelectric devices used only the history dependence of polarization switching to realize the short-term memory [2]; however, the memory effect may degrade or even disappear when the polarization is approaching saturation. This issue does not exist in our volatile FD because it also exhibits the spontaneous polarization back-switching induced by E_{imp} besides the history dependence of polarization switching. These complex polarization dynamics lead to a short-term memory effect with time constants in the millisecond scale. Accordingly, the pulse intervals used in the Hénon map prediction task are designed to match well with the time constants, ensuring the effectiveness of the short-term memory.

On the other hand, the nonvolatile FD-based readout network is also an important part of our RC system. The nonvolatile FD exhibits good polarization stability because of the absence of E_{imp}. Owing to this and the polarization-controlled conduction behavior, the

nonvolatile FD exhibits multilevel nonvolatile conductance states (>4 bits), which can be used to precisely map the weights in the readout network. This is the major contribution from the nonvolatile FD to the RC performance.

In short, the device characteristics contributing to the good performance in the Hénon map prediction and their physical mechanisms can be summarized as follows. 1) For the volatile FD for the reservoir, the nonlinearity and short-term memory are the most important device characteristics. The nonlinearity mainly originates from both the nonlinear polarization switching and the nonlinear polarization-controlled conduction behavior. In particular, the tiny and irregularly-shaped domains allow the nonlinear polarization switching to occur in a relatively wide range of voltages, covering the voltages applied in the Hénon map prediction task. On the other hand, the short-term memory mainly originates from both the history dependence of polarization switching and the spontaneous polarization back-switching induced by E_{imp}. The pulse intervals used in the Hénon map prediction task are designed to match well with the time constants of memory decay, ensuring the effectiveness of the short-term memory. 2) For the nonvolatile FD for the readout network, a sufficiently large number of conductance states with good retention are the most important device characteristics. Owing to the nonvolatility of polarization in absence of E_{imp} and the polarization-controlled conduction behavior, our nonvolatile FD exhibits multilevel nonvolatile conductance states (>4 bits), which can be used to precisely map the weights in the readout network.

Supplementary Note 4. Estimations of power and energy consumptions

We focused on the power and energy consumptions of the nonvolatile and volatile FDs, while those of the peripheral circuits were not considered. Taking the curvature discrimination task as an example, the write pulses applied to the reservoir have an average amplitude of ~–2.2 V and a width of 10 ms. The output currents of the volatile FDs in response to write pulses are ~–5.4 μA in average. The power and energy consumptions of the volatile FD are thus roughly estimated to be ~11.8 μW per input and ~118 nJ per input, respectively. Such power consumption is at least 3 times lower than those of the state-of-the-art filamentary memristors used for RC hardware systems [7-9]. However, the energy consumption of the volatile FD is relatively high, due to the large pulse width (i.e., 10 ms) as limited by our test board system. It is thus quite promising to boost the energy efficiency of the volatile FD by reducing the pulse width.

On the other hand, the input pulses applied to the readout network have an average amplitude of ~0.7 V and a width of 1 ms. The output currents of the nonvolatile FDs are ~200 nA in average. The power and energy consumptions of the nonvolatile FD are thus roughly estimated to be ~140 nW per input and ~140 pJ per input, respectively. Both the power and energy consumptions of the nonvolatile FD are very small, much lower than those of the volatile FD. Note that in the curvature discrimination task the nonvolatile FDs only performed inference after programming, and thus the programming energy was not considered.

References

1. Yu, J. et al. Energy efficient and robust reservoir computing system using ultrathin (3.5 nm) ferroelectric tunneling junctions for temporal data learning. In *2021 Symposium on VLSI Technology* 1-2 (2021).

2. Toprasertpong, K. et al. Reservoir computing on a silicon platform with a ferroelectric field-effect transistor. *Commun. Eng*. 1, 21 (2022)

3. Tang, M. et al. A Compact Fully Ferroelectric-FETs Reservoir Computing Network With Sub-100 ns Operating Speed. *IEEE Electron Device Lett*. 9, 43 (2022).

4. Liu, K. et al. Multilayer reservoir computing based on ferroelectric α-In_2Se_3 for hierarchical information processing. *Adv. Mater.* 22, 2108826 (2022).

5. Liu, K. et al. An optoelectronic synapse based on α-In_2Se_3 with controllable temporal dynamics for multimode and multiscale reservoir computing. *Nat. Electron.* 22, 1-13 (2022).

6. Li, W. et al. Polarization-Dominated Internal Timing Mechanism in a Ferroelectric Second-Order Memristor. *Phys. Rev. Appl.* 19, 014054 (2023).

7. Moon, J. et al. Temporal data classification and forecasting using a memristor-based reservoir computing system. *Nat. Electron.* 2, 480-487 (2019).

8. Zhong, Y. et al. Dynamic memristor-based reservoir computing for high-efficiency temporal signal processing. *Nat. Commun.* 12, 408 (2021).

9. Park, S. O. et al. Experimental demonstration of highly reliable dynamic memristor for artificial neuron and neuromorphic computing. *Nat. Commun*. 13, 2888 (2022).

10. Midya, R. et al. Reservoir computing using diffusive memristors. *Adv. Intell. Syst.* **1**, 1900084 (2019).

11. Liu, H. J. et al. Strain-driven phase boundaries in $BiFeO_3$ thin films studied by atomic force microscopy and x-ray diffraction. *Phys. Rev. B*. 85, 014104 (2012).

12. Chen, D. et al. Stripe domains in epitaxial $BiFeO_3$ thin films on (100) $SrTiO_3$ substrates. *J. Appl. Phys.* 123, 044102 (2018).

13. Pintilie, L., Boerasu, I., Gomes, M. J. M., et al. Metal-ferroelectric-metal structures with Schottky contacts. II. Analysis of the experimental current-voltage and capacitance-voltage characteristics of $Pb(Zr,Ti)O_3$ thin films. J. Appl. Phys. 98, 124104 (2005).

14. Pabst, G. W. et al. Leakage mechanisms in $BiFeO_3$ thin films. *Appl. Phys. Lett.* 90, 072902 (2007).

15. Pantel, D., Alexe, M. Electroresistance effects in ferroelectric tunnel barriers. *Phys. Rev. B.* 82, 134105, (2010).

16. Lee, D. et al. Polarity control of carrier injection at ferroelectric/metal interfaces for electrically switchable diode and photovoltaic effects. *Phys. Rev. B* 84, 125305 (2011).

17. Yang, H. et al. Rectifying current-voltage characteristics of $BiFeO_3$/Nb-doped $SrTiO_3$ heterojunction. *Appl. Phys. Lett.* 92, 102113 (2008).

18. Mocherla, P. S. V. et al. Tunable bandgap in $BiFeO_3$ nanoparticles: the role of microstrain and oxygen defects. *Appl. Phys. Lett.* 103, 022910 (2013).

19. Wang, C. et al. Switchable diode effect and ferroelectric resistive switching in epitaxial $BiFeO_3$ thin films. *Appl. Phys. Lett.* 98, 192901 (2011).

20. Kozakov, A. T. et al. X-ray photoelectron study of the valence state of iron in iron-containing single-crystal ($BiFeO_3$, $PbFe_{1/2}Nb_{1/2}O_3$), and ceramic ($BaFe_{1/2}Nb_{1/2}O_3$) multiferroics. *J. Electron Spectrosc.* 184, 16-23 (2011).

21. Li, C. et al. TiO_2 Coated polypropylene membrane by atomic layer deposition for oil–water mixture separation. *Adv. Fiber Mater.* 3, 138-146 (2021).

22. Bai, K. et al. Selenium nanoparticles-loaded chitosan/citrate complex and its protection against oxidative stress in D-galactose-induced aging mice. J. nanobiotechnology 15, 92 (2017).

23. Lu, Y. et al. Investigation of In-doped $BaFeO_{3-\delta}$ perovskite-type oxygen permeable membranes. *J. Mater. Chem. A*. 3, 6202-6214 (2015).

24. Cao, Y. et al. Phase-field modeling of switchable diode-like current-voltage characteristics in ferroelectric $BaTiO_3$. *Appl. Phys. Lett.* 104, 182905 (2014).

25. Pike, G. E. et al. Voltage offsets in (Pb, La)(Zr, Ti)O_3 thin films. *Appl. Phys. Lett.* 66, 484-486 (1995).

26. Fan, Z. et al. Ferroelectric diodes with charge injection and trapping. *Phys. Rev. Appl.* **7**, 014020 (2017).

27. Milano, G. et al. In materia reservoir computing with a fully memristive architecture based on self-organizing nanowire networks. *Nat. Mater.* 21, 195-202 (2022).

28. Du, C. et al. Reservoir computing using dynamic memristors for temporal information processing. *Nat. Commun*. 8, 2204 (2017).

29. Abreu Araujo, Flavio. et al. Role of non-linear data processing on speech recognition task in the framework of reservoir computing. *Sci. rep.* 10, 328 (2020).

30. Pintilie, L. et al. Ferroelectric polarization-leakage current relation in high quality epitaxial Pb(Zr, Ti)O_3 films. *Phys. Rev. B* 75, 104103 (2007).
