# Supplementary material for: All-ferroelectric implementation of reservoir computing
Source: Nat Commun. 2023 Jun 16;14:3585. doi: 10.1038/s41467-023-39371-y (PMC10275999; doi:10.1038/s41467-023-39371-y)
Supplement: Supplementary file 3 — Description of Additional Supplementary Files [file 41467_2023_39371_MOESM3_ESM.docx]

**Description of Additional Supplementary Files**

**File Name: Supplementary Movie 1
Description:** A video showing the operation of the all-ferroelectric reservoir computing system in the curvature discrimination task.
